# Advances in lithographic techniques for precision nanostructure fabrication in biomedical applications

**DOI:** 10.1186/s11671-023-03938-x

**Published:** 2023-12-11

**Authors:** Kate Stokes, Kieran Clark, David Odetade, Mike Hardy, Pola Goldberg Oppenheimer

**Affiliations:** 1 Advanced Nanomaterials Structures and Applications Laboratories, School of Chemical Engineering, College of Engineering and Physical Sciences, University of Birmingham, Edgbaston, Birmingham, B15 2TT UK; 2 School of Biological Sciences, Institute for Global Food Security, Queen’s University Belfast, Belfast, BT9 5DL UK; 3 Centre for Quantum Materials and Technology, School of Mathematics and Physics, Queen’s University Belfast, Belfast, BT7 1NN UK; 4 Healthcare Technologies Institute, Institute of Translational Medicine, Mindelsohn Way, Birmingham, B15 2TH UK; 5 Cavendish Laboratory, Department of Physics, University of Cambridge, JJ Thomson Avenue, Cambridge, CB3 0HE UK

## Abstract

Nano-fabrication techniques have demonstrated their vital importance in technological innovation. However, low-throughput, high-cost and intrinsic resolution limits pose significant restrictions, it is, therefore, paramount to continue improving existing methods as well as developing new techniques to overcome these challenges. This is particularly applicable within the area of biomedical research, which focuses on sensing, increasingly at the point-of-care, as a way to improve patient outcomes. Within this context, this review focuses on the latest advances in the main emerging patterning methods including the two-photon, stereo, electrohydrodynamic, near-field electrospinning-assisted, magneto, magnetorheological drawing, nanoimprint, capillary force, nanosphere, edge, nano transfer printing and block copolymer lithographic technologies for micro- and nanofabrication. Emerging methods enabling structural and chemical nano fabrication are categorised along with prospective chemical and physical patterning techniques. Established lithographic techniques are briefly outlined and the novel lithographic technologies are compared to these, summarising the specific advantages and shortfalls alongside the current lateral resolution limits and the amenability to mass production, evaluated in terms of process scalability and cost. Particular attention is drawn to the potential breakthrough application areas, predominantly within biomedical studies, laying the platform for the tangible paths towards the adoption of alternative developing lithographic technologies or their combination with the established patterning techniques, which depends on the needs of the end-user including, for instance, tolerance of inherent limits, fidelity and reproducibility.

## Introduction

The ability to manufacture nano- scale components has enabled the production of highly capable devices, generating technological advancements in many industries. Progress in lithographic techniques often centres around semiconductor research and the ever-decreasing size of transistors—the miniature components that can alter electrical signals—and consequently increasing number being squeezed into computer chips to form logical components to make decisions at the nano-level. The importance of continued transistor research has been underlined recently by spending in the US and EU, where the US Government signed off a $280bn package, the CHIPS and Science Act, to stimulate Stateside chip growth, while the EU has proposed doubling their chip production by 2023 with a $50bn spend. Elsewhere, the Taiwan Semiconductor Manufacturing Company have resolved to keep apace with Moore’s law by opening a new microchip factory at a cost of $33bn in 2025. However, not all progress is exclusively focused on computer processing power. For instance, in the healthcare industry, miniaturisation has led to developments such as minimally invasive surgery with laparoscopic cameras [1], lab-on-a-chip technologies for point-of-care diagnostics [2] and improved implantable devices [3]. Other sectors which have benefitted from these advancements include energy [4, 5], communications [6, 7] and sensing [8, 9].

Lithography is a fabrication technique which enables the patterning of structures on a substrate [10]. Two of the most common lithographic techniques are photolithography (PL), which involves applying light through a mask onto a photosensitive resist to generate structures [11] and electron beam lithography (EBL), where electrons scan a surface covered in a sensitive resist, with the electron beam turning on and off to produce structures in the desired locations [12, 13]. Table 1 provides an overview of the most common conventional lithographic techniques [12–26]. To date however, there are challenges with the existing methods for instance, related to resolution, position control, physical limits, versatility, reproducibility, and scalability, exacerbated by the inability of flexible patterning of different materials for sufficient throughput for commercial applications [17, 20, 21, 28]. Importantly, depending on the application, the requirements for the optimal patterning process may vary. While most micro-to-nanofabrication techniques utilise resists of macromolecular nature, the extension towards additional material systems such as glassy, ceramic, ferroelectric and conductive materials is of major importance, and cannot be accomplished with resists alone. Moreover, for many applications it is desirable to control the spatial arrangement of more than one component, relative to other elements within the pattern. With traditional methods, the process requires an iterative multistep procedure, rendering the patterning process intricate and resulting in a reduction of yields and reproducibility.

**Table 1:** *S*ummary of conventional lithographic techniques and their advantages and limitations

Technique	Brief summary	Resolution	Advantages	Disadvantages
UV lithography	Use light and mask to pattern a photoresist [27]	1 µm [28]	Simple [15] Efficient [15] Parallel processing allows mass production [29]	Clean room required [30] Resolution limited by diffraction [31] Substrate must be flat [30]
Deep UV lithography	Use UV light and mask to pattern a photoresist. Wavelength reduced to 193 nm or 248 nm [32]	65–130 nm [32]	Improved resolution in comparison to traditional UV lithography [32]	Shorter wavelengths are more easily reflected [33] Interference effects [32] Maximum total thickness variation is 0.5 µm [34] Low depth of focus [34]
Extreme UV lithography	Use UV light and mask to pattern a photoresist. Wavelength reduced to 13.5 nm [35]	< 10 nm [36]	Improved resolution in comparison to traditional UV lithography [36]	Shorter wavelengths are more easily reflected [33] Low photon transmission efficiency [37] Defects in the photomask warp pattern [38] Secondary electrons cause blur [39] Increased stochastic pattern variations [40]
X-ray lithography	Use x-rays and mask to pattern a photoresist [41]	15 nm [42]	Improved resolution in comparison to traditional UV lithography [42] Large substrate-mask distances do not cause diffraction/proximity effects until the feature width approaches 100 nm [43]	Shorter wavelengths are more easily reflected [33] Masks are thin, fragile, and expensive [44] Secondary electrons cause blur [45]
Electron beam lithography	Use electrons to pattern a resist [46]	> 10 nm [47]	Precise control [48] Can pattern complex geometries [48]	Beam can damage the substrate [20] Proximity effect generates unwanted patterns/distortions [19] Complex and costly [20] Low throughput [20]
Focused ion beam lithography	Uses ions to pattern a resist [49]	> 10 nm [50]	Proximity effect, forward scattering and radiation damage are reduced in comparison to EBL [20, 46]	Ion beam can damage the sample [22] Sputtering creates unwanted defects [22] Slow – the time required is dependent on the milling area [22]
Soft lithography	Uses an elastomeric mould (stamp) to fabricate and replicate structures [23]	35 nm [51]	Substrate flatness is not as critical [24] Can include biomaterials [24]	Mould known as a master must be fabricated to produce the stamp [24] Must be carried out in a clean room [25] Large start-up costs [25] Removing stamp from the master can cause damage [25]
Scanning probe lithography (SPL)	A sharp tip changes the structural or chemical properties of the surface it is in contact with	4–10 nm [52]	Cost-efficient [26] Can be carried out in ambient conditions [26] Substrates do not require further development [26]	Low throughput – can be improved by using multiple tips in parallel [21]

The growing demand for better performance, reduced energy consumption, high-throughput, with higher levels of complexity and ease-of-integration has raised the need for alternative techniques capable not only of the generation of patterns below the sub-micrometre scale but which are flexible, scalable and low cost. This has introduced many manufacturing challenges. The unique properties of the many nanostructured platforms and their potential revolutionary applications remain a challenge to implement in practice due to the lack of sufficient single-step processes, which can integrate technologies across several orders of magnitude of size as well as enable large-scale manufacturing. As demand for smart multifunctional devices continues to grow, manufacturing solutions are needed to address nano-patterning requirements that cannot be supported by conventional fabrication, and which can be easily scalable from die-level all the way up to large-area substrates. Thus, development of cost-effective lithographic processes to enable straightforward high-fidelity patterning in a controlled manner on multiple scales, which will also be suitable for a broad range of functional materials with embedded scalability are essential for the successful future large-scale manufacturing of advanced technologies to accomplish the industrial requirements with excellent capability to meet future challenges.

In the past decades, while aiming to overcome the various challenges and limitations of conventional lithographic techniques, new fabrication methods have been developed, broadly titled emerging lithographic techniques [53–55]. Herein, we firstly outline and summarise the most common conventional lithographic techniques along with the associated challenges, highlighting the need for the development of new fabrication methods and subsequently, overview the emerging patterning techniques (Table 2), whilst describing their basic principle and comparing lithographic performance in terms of resolution, speed, cost as well as advantages and disadvantages in comparison to the most common conventional lithographies, providing an overall assessment of their potential to overcome the current challenges in the microfabrication industry towards generating further technological advancements.

**Table 2:** Overview summary of emerging lithographic techniques

Technique	Brief Summary	Resolution	Advantages	Disadvantages
Evanescent near-field optical lithography	Use UV light and mask to pattern a photoresist. Mask and substrate separated a distance less than the wavelength of incident light [326]	26 nm [156]	Low cost [159]	Must use flexible photomasks [158] Exposure of unwanted areas of the substrate [158] Depth of field reduces as structure size decreases [158]
Immersion lithography	Use UV light and mask to pattern a photoresist. Transparent liquid is placed in between the optical apparatus and photoresist [71]	38 nm [165]	Improves photolithography resolution [165]	Fluid must meet specific requirements [167] Contaminants from fluid must be removed [167] Photoresist–liquid interactions must be well understood [167]
Stereo- LITHOGRAPHY	Cures a photopolymer resin layer-by-layer [172]	0.6–10 µm [175, 176]	Can generate complex 3D structures over a large area [327]	Limited to specific photopolymers [177] Must be efficiently ventilated [179]
Two-photon lithography	Uses UV light to pattern a substrate via two-photon polymerisation [186]	150 nm [187]	Use of two photons enables smaller dimensions can be generated [187] Able to generate 3D structures [186]	Time-expensive for large structures [185] Large amount of data storage required to digitally define the pattern [185]
Block copolymer lithography	Links together two immiscible monomers which separate into microdomains [194]	< 10 nm [197]	Low cost [195] Simple [195] Precise [195] Can cover large areas [195]	One monomer may be more likely to wet the substrate [198] Periodicity of microdomains limits patterning possibilities [193, 199]
Nanosphere lithography	A material is patterned with a colloidal crystal mask (CCM), produced from nanospheres [207]	1 nm [219]	Relatively low cost [218] Can create versatile, controllable periodic structures [218, 222] Fabricated structures have dimensions ~ 1/5 size of the nanospheres [219]	Restricted pattern geometries [220] Difficult to control crystal domain size, defects, and dislocations [220]
Nanoimprint lithography	Polymer is spin-coated onto a substrate and a stamp is pushed down onto it [240]	< 3 nm [253]	Precise [218] Inexpensive [218] High throughput [218] Can produce nanoscale features over a large area [218]	Interactions between the stamp/polymer/substrate can cause incomplete pattern replication or defects [254]
Edge lithography	Uses topographic edges to pattern arrays [265]	< 100 nm [53]	Can increase the nanofabrication efficiency, strength, shape, resolution, and cost of other lithographic techniques [265, 266]	Can only pattern relatively small areas [53]
Electrohydrodynamic lithography	Uses an electric field to destabilise a thin-film polymer to form polymer pillars [275]	< 50 nm [278]	Simple [277] Low cost [277] Versatile [277] Can combine different materials into polymer pillars [279]	Pillars may be non-uniform with rough edges [281] Fabrication time must be carefully selected [282] Fabrication times can be long if high-viscosity polymers are used [282]
Capillary force lithography	Polymer is spin-coated onto a substrate, a stamp is placed on it, and heated and then cooled below the polymer’s glass transition temperature [287]	< 100 nm [287]	Versatile [287] Generates precise features [287] Performed under ambient conditions [290]	Aspect ratio must be carefully selected [287] Creates low-density patterns [287]
Near-field electrospinning-assisted lithography	A polymer is deposited from a tip to the substrate via an electric field. It is then coated with a scaffold material and the original polymer is removed [293]	1 $\upmu$ m width, 65 nm depth [293]	Simple [293] Efficient [293] Low cost [293] Nanometre-scale depths [293]	Challenging to pattern large areas [293] Requires two polymers and a non-toxic solvent which only removes one of these polymers [293]
Magneto lithography	Uses ferromagnetic nanoparticles to either directly pattern a surface or to block active sites when the target material is applied [301]	< 100 nm [297]	Inexpensive [297] High throughput [301] Lack of resist decreases surface contamination [297] Can pattern curved surfaces [297] Environmental conditions can be carefully monitored and controlled [297] Fabrications can be smaller than the mask [297]	Nanoparticles tend to migrate and clump together [297] Must be a balance between uniformity of structures and cost [297]
Magneto-rheological drawing lithography	Produces microneedles arrays using droplets of curable magnetorheological fluid and an external magnetic field [307]	600–700 µm height [308, 309] 185–650 µm width at base [308, 309] 10–15 µm width at tip [309]	Cost-effective [310] Simple [310] Rapid [310] Easily controlled with external magnetic field [311]	Limited to magnetic materials [311] Limited applications as iron particles are toxic [311]
Nanotransfer printing	Uses a stamp to transfer a pattern from one substrate to another [248]	< 10 nm [317]	Able to generate 3D structures [315] Low cost [328] High throughput [315] Can produce structures on flexible and non-planar substrates [328]	Time-consuming [319] Can only be carried out over small areas [319]

## Conventional lithographic techniques

### Photolithography

Photolithography (PL) is a top-down fabrication technique in which a substance known as a photoresist is exposed to light in certain regions, which can be further developed to create the desired product [11, 56]. Photoresists are materials sensitive to UV light and are generally composed of polymers, which change structure in the presence of UV radiation, sensitisers, which determine the solubility of the photoresist and solvents, which change the viscosity enabling easy applicability onto the desired substrate [57]. Two main categories of photoresists include the positive photoresists, which become soluble in presence of UV light and the negative photoresists, which become insoluble [27]. The photoresist is typically deposited in the middle of a wafer, commonly coated with an oxide layer, which acts as a barrier against diffusion of impurities [58]. It then undergoes spin-coating, which forms a thin uniform layer on a wafer, the depth of which is inversely proportional to the square root of the rotation speed [59]. The wafer is later soft baked to remove any excess solvent, stabilise the photoresist and improve its adhesion [60, 61]. After cooling, a pattern is formed on the wafer via a photomask [62], which is opaque to UV light, with various transparent areas allowing transmission through. Photomasks, aligned with the wafer using a mask aligner or fiducial marks, with the UV light interacting with the photoresist, produce the desired pattern on the wafer [63, 64].

#### Conventional UV lithography

Conventional UV lithography (UVL) utilises wavelengths between 436 and 356 nm to pattern the photoresist. UVL is a simple and cost-effective technique that allows parallel processing, such that many patterns can be produced in a relatively short period of time [14, 15]. However, the substrate must remain flat, otherwise the planar photomask would only be in contact with, or focus the UV light onto, a small area of the substrate [18]. Furthermore, as any surface impurities can create defects in the final lithographic pattern and impede its functionality, the substrates must be thoroughly cleaned to remove surface impurities and often necessitates a clean room environment [16, 65].

The choice of photoresist also must also be carefully considered. Negative photoresists can expand as they develop, deforming the resultant pattern [66] and while positive photoresists offer a higher resolution, they come with a higher cost and lower adhesive properties. Consequently, it might be necessary to apply an even monolayer of an adhesion promoter like hexamethyldisilazane (HMDS) onto the substrate. [27, 67]. Moreover, although soft baking is required as solvent evaporation can alter the photoresist’s properties, it can also cause negative effects such as sensitiser decomposition and therefore, the parameters must be optimised to maximise the quantity of evaporated solvent whilst decreasing the amount of decomposition [60].

However, arguably the biggest restriction is the resolution, which, predominantly, is diffraction-limited to approximately 1 µm [16, 28]. Various methods have been developed to improve the resolution of UVL, such as evanescent near-field lithography (ENFOL), which involves changing the distance between the wafer and the light source [68–70], immersion lithography (IL), where the refractive index between the light source and the photoresist is increased [71]. These are discussed further in the ‘Emerging Lithographic Techniques’ section. Alternative methods such as deep UV lithography (DUVL), extreme UV lithography (EUVL) and x-ray lithography (XRL) are detailed below and involve reducing the wavelength of the light [35, 72, 73].

Conventional UV lithography finds applications in diverse fields, including advanced integrated optoelectronic devices, multifocal microlens arrays for arrayed cameras and high-resolution quantum dot light-emitting diodes for display technologies [74–76]. Its widespread availability, high throughput and capacity for producing structures over large areas enables UVL to be employed to create various structures for biomedical devices [77]. For example, Rüegg et al. [78] used UVL, alongside physical vapour deposition and ion beam etching, to generate biodegradable frequency-selective magnesium radio-frequency microresonators for transient biomedical implants. Recently, Wu et al. [79] used UVL to produce hollow large arrays of microneedles. These microneedles offer a promising method for collecting and monitoring biomarkers in skin interstitial fluid, providing an alternative to traditional blood sampling.

However, the cytoxicity of free radicals generated during the absorption of incident light by the photoinitiator poses a challenge to cell viability. Furthermore, to create multilayer structures such as tissue scaffolds using photolithography, multiple distinct photomasks are essential, leading to an increased prefabrication time, costs and complexity. The photomasks must be manually changed and aligned after each layer, underscoring the need for research in automation techniques to streamline this process [80].

#### Deep UV lithography

Although a very similar process to UVL, the key difference between UVL and deep UV lithography (DUVL) is the wavelength of the UV light. DUVL operates at shorter wavelengths than UVL, typically between 193 and 248 nm [72], which results in resolutions of between 65 and 130 nm [32]. To generate the intensity and wavelengths required, DUVL utilises quartz lenses and excimer lasers to produce shorter UV wavelengths, wavelengths of 193 nm and 248 nm are produced using gas chambers of ArF and KrF respectively [81, 82]. Shorter light wavelengths are reflected more easily by the silicon wafer and consequently this reflected light from the wafer can also react with the photoresist, causing widespread deformities or localised defects [33]. Furthermore, control over the critical dimension is limited by the reflectivity. The combination of the reflectivity and internal reflections due to a difference in refractive index at the air-photoresist boundary can generate a standing wave in the photoresist, creating significant constructive or destructive interference effects [32]. Therefore, a polymer-based bottom antireflective coating is applied to the wafer, which absorbs the UV light that has passed through the photoresist and reduces negative reflective effects [83].

Another variation between UVL and DUVL is the required flatness of the wafer, which is often measured by analysing the total thickness variation (TTV) [84]. The depth of focus (DOF) gives the system tolerance to differences in surface height, including effects such as TTV, and is proportional to the wavelength of light. Consequently, the most precise UVL processes require a TTV of 2 µm across the wafer, whereas this is 0.5 µm for DUVL [34], and therefore DUVL wafers must undergo additional chemical mechanical polishing and subsequent cleaning to remove any residual material to produce the TTV required [32]. Due to the low depth of field (DOF), the DUVL photoresist must be thin and highly sensitive to DUV wavelengths and so chemically amplified photoresists are employed. Typically these take advantage of photoacid generators (PAGs) which produce an acid when interacting with a UV photon, generating a cascade of chemical reactions and increasing the effective quantum efficiency [85], enabling features smaller than the UV wavelength to be fabricated [86]. The resists are temperature-sensitive and therefore temperature variation across the hot plate during the post-baking process must be carefully monitored to prevent pattern warping [87]. Defects in the photoresist can also cause patterning issues and therefore defect detection and removal is an essential step in fabrication [88].

**Fig. 1:**
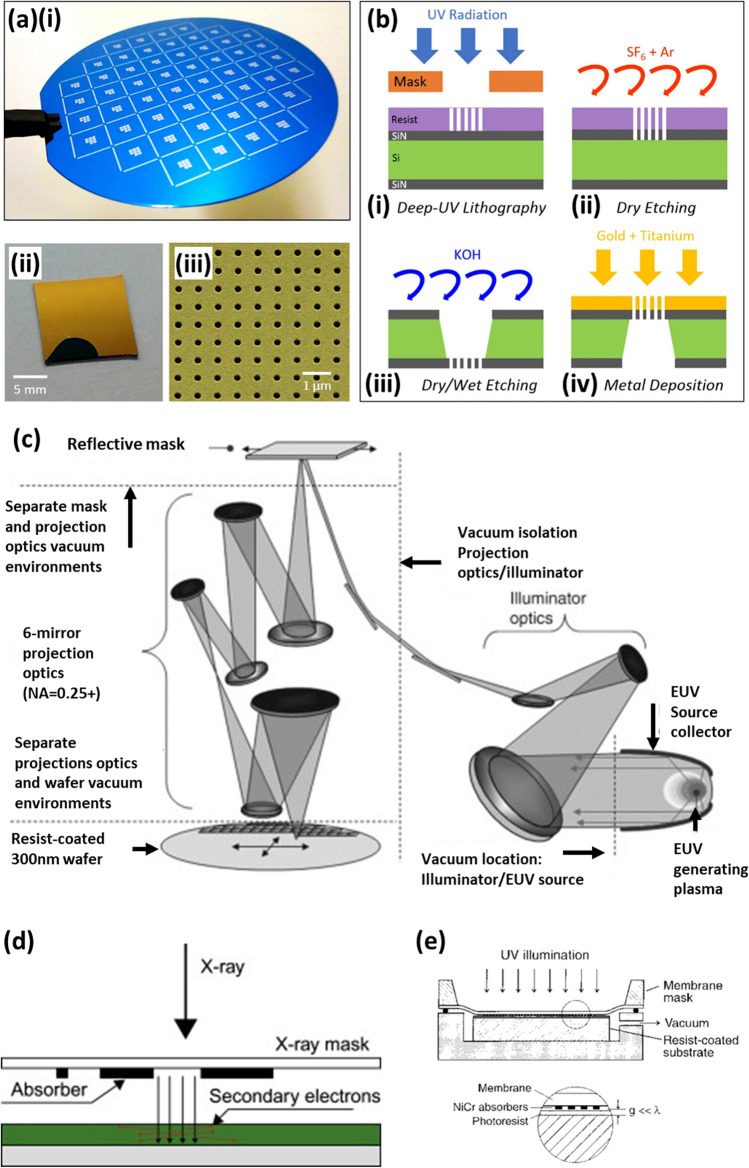
**a(i)** Photography of the wafer after deep-UV and dry etching steps. **(ii)** Single plasmonic chip containing eight plasmonic pixels. **(iii)** SEM image of the nanohole array with a hole diameter of 200 nm and an array period of 600 nm. **b** Fabrication steps of the nanohole arrays on free-standing silicon nitride membrane **(i–iv)**. Image from [91]. **c** Schematic illustration of extreme ultraviolet lithography process. Adopted with permission from [99]. **d** Diagram of x-ray lithography (adopted with permission from [42]). **e** Schematic diagram illustrating the evanescent near-field optical lithography (ENFOL) process. Image from [89]

DUVL has proven useful in a wide range of applications. Dumon et al. and Bogaerts et al. demonstrated the use of DUVL to fabricate photonic wires which could lead to greater photonic integration, producing small devices with improved optical capabilities at a much lower cost [89, 90]. The high resolution, high throughput and low cost of DUVL has also lended itself to biomedical applications. Coskun et al*.* [91] developed plasmonic chip for label-free biosensing, as shown in Fig. 1a. The fabrication process (Fig. 1b) involved a combination of DUVL, etching and metal deposition. Furthermore, Van Gerwen et al. [92] generated nanoscale electrodes with enhanced sensitivity for the identification of viruses and genetic conditions, by measuring the change in electric field when the antibodies or DNA interacts with antigens or probes respectively. Elsewhere, Wang et al. developed a molecular sentinel-on-chip device fabricated with techniques such as DUVL to recognise DNA through surface-enhanced Raman spectroscopy (SERS). This device was able to indicate whether the Ki-67 gene, a biomarker for breast cancer, was present within the DNA, and could be used in screening and diagnosis of further medical conditions [93].

DUVL faces analogous challenges in the biomedical industry as UVL and in addition, the stringent requirements for DUVL escalate the cost, necessitating a compromise between resolution and expense [94]. Moreover, with extreme UV lithography capable of achieving higher resolutions, the applicability of DUVL may become outdated [32].

#### Extreme UV lithography

DUVL can be advanced further with extreme UV lithography (EUVL), which produces nanoscale features with soft x-ray photons with a wavelength of 13.5 nm, generated from a plasma source such as laser-produced transient tin plasmas [35]. As shorter wavelengths are more easily absorbed by materials, EUVL must be carried out in a vacuum [95]. The photons travel through a series of multilayer mirrors, consisting of alternating layers of material with high and low atomic numbers, such as molybdenum and silicon, producing near-normal incidence with a reflection efficiency of approximately $70\mathrm{\%}$ [96]. Unlike DUVL, the EUVL photomasks also reflect rather than block portions of light from interacting with the photoresist [97]. The photomasks are produced from a glass substrate with a low thermal expansion, which reduces the possible distortion of the photomask and is coated with a multilayer reflective material, such as silicon and molybdenum, and a layer of absorbing material such as chrome [98]. The image reflected from the photomask is de-magnified and focused onto the photoresist by another series of multilayer mirrors. The typical configuration for EUVL is shown in Fig. 1c [99].

EUVL is able to produce sub-10 nm structures, however it does present a number of challenges [36]. Defects in the photomask, for example pits and small particles added during its production, can alter its reflective properties and therefore, warp the pattern fabricated in the photoresist [38]. Most defects exist on the substrate and therefore focus must be given to substrate cleaning. These defects include soft particles bonded to the surface via van der Waals forces, and hard particles which can be embedded in the substrate and leave a pit when removed [100]. Various pit smoothing techniques have been devised [101], however, these methods are limited by the ability to analyse the substrate to identify the defects which must be removed [102, 103]. Furthermore, the compound effect of multiple mirrors results in low efficiency, with approximately 1–5% of the photons produced from the UV source interacting with the wafer. Consequently, a high-power EUVL laser is required to compensate for this loss and the photoresist must have a high sensitivity to detect and react to the low throughput of these photons [37, 39]. Due to the high energy of the photons, the interaction between photons and the resist can create photoelectrons which scatter to form secondary electrons, these can be re-absorbed by the resist and cause unwanted areas of exposure known as blur, which limits the resolution of EUVL [39]. Moreover, the low throughput of photons leads to the enhanced contribution of photon shot noise, resulting in increasing stochastic pattern variations [40]. However, there is continued research into overcoming these issues, for example by producing alternative high power light sources [104], improving the conversion efficiency from laser energy to EUV radiation [105, 106] and reducing defects through improved photoresists [107, 108] and coatings such as stochastic defect removal coatings [109].

EUVL, which is dominated by Dutch firm ASML selling systems at $150 m, has enabled denser integrated circuits to be produced which demonstrate speeds up to 100 times greater than the current most advanced chips [110]. Machines manufactured by advanced semiconductor materials lithography have achieved resolutions of 13 nm and can produce 3 nm, 5 nm and 7 nm logic nodes; developments are currently underway to produce 2 nm logic and memory nodes by increasing the size of the numerical aperture [40, 111]. Apple have recently used EUVL to generate their A14 processor with 5 nm process nodes, while Google has used the increased power provided by EUVL components to improve search engine results, and further integration of EUVL in industry could lead to improvements in memory chips, CPU chips and integrated circuits for 5G communications [112]. Furthermore, the potential to create devices with advanced capabilities with EUVL could greatly benefit the biomedical industry, enhancing biosensors for the remote monitoring of patient health and improving artificial intelligence for applications such as disease prediction and diagnosis [113–115].

The cost and complexity of EUVL may constrain its application in the mass production of biomedical devices. The stringent demands for defect-free reflective photomasks in particular present challenges to its widespread adoption. Nevertheless, with anticipated technological advances aiming to enhance simplicity and reduce costs in these systems, the resolution remains advantageous for biomedical applications such as nanofluidics [116, 117].

#### X-ray lithography

X-ray lithography (XRL) (Fig. 1d**)** [42] is a similar process to UVL except for the UV light being replaced with x-rays created by a synchrotron source, with wavelengths between 0.4 and 4 nm [73]. The amount of x-ray absorption is dependent on the thickness, density and the atomic number of the material it is travelling through [118]. Therefore, x-ray masks are produced from thin layers of low-atomic materials to allow x-ray transmission, integrated with regions of high atomic number materials which prevent transmission, creating a pattern of x-rays which is transferred onto the resist. Examples of materials for x-ray masks include transmission materials such as silicon, silicon carbide and silicon nitride, and an absorber material such as gold [119]. Due to the specific requirements, producing the x-ray masks is problematic and the resolution required determines the manufacturing process. Micrometre resolution can be achieved by depositing gold on the x-ray transparent substrate via methods such as sputtering, electroplating or thermal/electron beam evaporation. The gold can then be patterned, using techniques such as UVL and subsequent etching, to form the final mask. To produce masks with a higher resolution an intermediary step must be implemented, involving soft XRL and masks produced with UVL or EBL [120]. However, x-ray masks are expensive to produce and are thin and fragile, so are easily warped by radiation damage [44].

XRL can achieve resolutions of approximately 15 nm [42]. Unlike methods such as UVL, XRL can cope with large proximity gaps without causing diffraction or proximity effects until the required feature size reaches approximately 100 nm [43]. However, like EUVL, the short wavelength and consequently high energy of the photons cause blur, which limits its resolution [45]. X-ray lithography has been used in several different technological applications. For example, Mazhar et al. generated grid dielectric resonator antennas using XRL which have much wider bandwidths and are more efficient for millimetre wavelengths, thus more data can be transmitted [121]. Moreover, Ryu et al. [122] investigated improving the efficiency of plasma display panels using barrier ribs cells with a high aspect ratio to enhance the phosphor layer and discharge space within the panels.

Jeong et al. demonstrated that XRL could also be advantageous in the medical industry. Certain drugs must be taken regularly, leading to patients having a strict medication schedule they must adhere to. The authors employed XRL to fabricate biodegradable polymer microstructures with polycaprolactone (PCL) which can release specific doses of medication into the patient’s system over time. This could be mass-produced and used for molecular targeted therapy, an alternative to chemotherapy without the drawbacks of toxicity and non-selectivity. X-ray lithography addresses several challenges that other techniques encounter. For instance, microstamping and cutting may struggle to achieve the precise shape patterning required due to issues like internal stress and thermal damage, and PCL cannot be patterned using UV lithographies [123].

One specific area that is benefitting from lithographic advancements is that of microfluidics. Microfluidics refers to micrometre-scale devices with liquid-phase flow and is a growing area that permits easy operation and portability for point-of-care technologies, which can also reduce the amount of analyte required, facilitate on-chip processing for full lab-on-chip implementation and thus, may aid the adoption of novel biofluids in medical diagnostics [124]. Mondal et al. produced microfluidic devices with a micromold generated with XRL which could be used in the medical industry and for environmental applications. XRL was selected for its capability to generate accurate microstructures with minimal surface roughness, a crucial requirement for effective microfluidic devices [125]. However, XRL may not be suitable for the mass production of biomedical systems. The high cost associated with investing in a synchrotron source and producing complex x-ray masks, coupled with the relatively low throughput, restricts its widespread use in the biomedical industry [41, 45].

### Electron beam lithography

Electron beam lithography (EBL) is a similar process to photolithography, except that it is a maskless technique [126]. EBL is *a top-down* approach, which utilises either a positive or negative resist, although its solubility is affected by exposure to a beam of electrons rather than UV light [12]. The electron beam scans the wafer in two different ways including, raster and vector scanning. During raster scanning, the wafer is divided into pixels and the beam scans each horizontal line from left to right. It then returns to the left-hand side of the wafer where it scans a new line until having analysed the entire surface, only switching on when the exposure is required. In a vector scan, the image is split into vectors, which contain the features and, instead of scanning the entire wafer, the electron beam moves from feature to feature [13]. The photomasks consist of a chrome layer deposited on a quartz substrate where the chrome layer is etched to create the pattern, which will be transferred to the photoresist, whilst the quartz substrate allows the electron transmission [127].

EBL enables precise control over the patterning of complex sub-micron geometries [48]. The advantage of EBL over PL is its ability to reach sub-10 nm resolution, due to the wavelength of electrons being much shorter than photons [47]. Considerations however, must be made regarding the energy of the electron beam, since accelerated electrons can achieve a higher resolution with less forward scattering yet are more likely to cause significant damage to the substrate [128].

The main drawback of EBL is the proximity effect. When the resist is exposed to the electron beam, the incident electrons can interact with electrons and atoms in the resist, deflecting the electrons at a small angle and generating forward scattered electrons. Electrons can be deflected at a larger angle by the substrate, producing backscattered electrons. This scattering of electrons leads to unintended exposure of the resist in regions not directly exposed to the electron beam, causing the dimensions of the developed patterns to deviate from the target dimensions [19]. Moreover, EBL is complex and costly, requiring a significant level of maintenance whilst often exhibiting a low throughput given its operating at a speed approximately ${10}^{7}$ times slower than optical lithography methods. This becomes especially significant when generating large and dense intricate patterns [20].

The nanoscale resolution offered by EBL holds significant promise in the biomedical industry. For instance, Vinje et al. utilised EBL to create nanostructured surfaces tailored for cell studies. This involved fabricating nanopillars with varying heights on a single substrate, which is challenging with alternative fabrication techniques [129]. Furthermore, Jiang et al. recently employed EBL to produce two-dimensional magnetic achiral nanorobots. The nanorobots demonstrated both biocompatibility and propulsive capabilities, offering a potential route for targeted drug delivery [130]. However, the high cost and low throughput of EBL still limit its mass production capabilities, thereby restricting its commercialisation generating medical devices [117].

### Focused ion beam lithography

Focused Ion Beam lithography (FIBL) is a *top-down* fabrication technique, which employs an ion beam to mill into the surface of a material and uses a polymethylmethacrylate (PMMA) resist [131]. The most common ion source is Gallium (Ga) due to its high atomic mass and low vapor pressure, melting point and volatility [132]. A liquid reservoir of Ga is positioned at the top of the FIB column and used to wet a sharp Tungsten tip. A high voltage creates a high electric field, which causes the Ga to form a sharp cone-like shape. The Ga atoms are then ionised by the electric field and the high voltage accelerates these ions down the column [49]. The Ga ions bombard the surface of the target material and displace the surface atoms, milling the surface and creating a pattern as the ion beam is moved along the surface [21].

Both EBL and FIBL offer sub-10 nm resolution, although FIBL offers many advantages over EBL [50]. For instance, the proximity effect is significantly reduced as ions are much heavier than electrons, decreasing the amount of back scattering. The amount of forward scattering is also reduced since the heavier ions have more momentum than electrons; the combination of the higher mass and momentum of the ions decreases the beam wavelength in comparison to an electron beam, drastically decreasing the amount of diffraction and increasing the resolution [20]. Furthermore, structures produced with FIBL are subjected to less radiation damage than EBL, due to FIBL having a high exposure sensitivity (with only 1–10% of particles being required in comparison to EBL to expose the resist) and the resist absorbing the majority of the ions [13].

However, FIBL can still damage the sample within proximity to the ion beam and metal atoms which have been sputtered from the sample can be redeposited in other areas, creating unwanted defects or contaminants. Unlike EBL, the length of the FIBL process is dependent amount of milling required and therefore, for large arrays, FIBL may not be the best suited fabrication method [22].

Md Ibrahim et al. employed FIBL to generate graphene nanohole/silicon micro-nanopore structures for DNA detection. The graphene served as the sensing membrane, while FIBL enabled conical-shaped micro-nanopore structures to be produced in the silicon. These structures acted as fluidic channel and enhanced the transport efficiency in microfluidic chips compared to cylindrical shaped pore structures [133]. Additionally, Mahajan et al. utilised FIBL to engineer nanostructures on implantable intracortical microelectrodes (IMEs). IMEs enable the monitoring of neural brain activity within the cerebral cortex, offering invaluable insights into neurological function and diseases. Additionally, they find applications in brain-machine interface systems for prosthetics and therapeutics. The nanostructured surface of the electrode holds promise for reducing neuroinflammation and enhancing neuronal viability. The capability of FIBL to directly fabricate nanostructures onto microelectrodes post-manufacture opens avenues for modifying and optimising medical devices after production [134, 135].

### Soft lithography

Soft lithography utilises an elastomeric mold known as a stamp to fabricate and replicate structures, with resolution of approximately 35 nm achieved via processes such as molding, printing and embossing [23, 51]. A polymer is poured into a previously fabricated mold, known as a master. Typically, poly(dimethylsiloxane), also known as PDMS, is exploited for this purpose, which is chemically inert, durable, relatively cheap and has low toxicity [51]. Since PDMS is elastomeric it can be cured and removed from the master to produce the stamp [136]. Using soft lithography, a surface can be patterned via different techniques, including:

- Microcontact printing—involves submerging the stamp into a solution and then placing it onto a substrate to transfer the pattern [137].
- Microtransfer molding—the stamp can be reversibly attached to a surface to construct microchannels, enabling fluid flow through the device. The fluid can deposit layers of material on the substrate, or it can undergo the curing process which converts the liquid into a solid. The PDMS can be removed to form the final pattern on the substrate [138].
- Replica moulding –prepolymer is poured over the stamp and placed on a substrate. This is subsequently, cured and the stamp is removed to produce a replicate pattern on the substrate [139].
- Solvent-assisted microcontact molding (SAMIM) – a solvent is applied to the stamp, wetting the surface. The stamp is then placed onto a polymer, where some dissolves and conforms to the mold profile. The solvent evaporates and the polymer re-solidifies, creating a negative version of the original stamp [140].
- Micromolding in microcapillaries (MMIC) – the stamp is positioned on a substrate to create microchannels. Capillary action causes the channels to fill with low-viscosity liquid when the fluid is situated near the end of the microchannel. Curing then builds microchannels on the substrate [141].

Soft lithography enables creating structures on non-planar surfaces and can include biological materials. However, it requires the production of a master via a technique such as photolithography, for each individual pattern [24]. It also often requires a clean room environment, large and expensive equipment as well as the taking into a consideration the challenges of removing the PDMS from the master, which can damage and introduce artifacts in the patterned structures [25].

The cost-effective production of flexible and biocompatible microchannels has positioned soft lithography as a versatile tool in the biomedical field to produce microfluidic chips and microelectromechanical systems (MEMS) for research, diagnostic and therapeutic applications [142]. For instance, Guo et al. [143] used soft lithography to fabricate a stretchable conductive microfluidic device for the study of cellular physiology and Zhang et al. [144] employed soft lithography to generate liquid metal-based soft microfluidic devices for the development of thermochromic sensors and flexible glucose biosensors.

The application of soft lithography in the biomedical industry is limited by the low bio-resistance of PDMS, which absorbs biomaterials and consequently, warps the PDMS and diminishes the efficiency of microfluidic biosensor systems. However, the introduction of new materials such as the high fluorinated elastomers or blending PDMS with curing agents, have been demonstrating a potential to reduce this impact [117].

### Scanning probe lithography

Scanning probe lithography (SPL) encompasses a group of techniques in which a small probe, such as the tip utilised in atomic force microscopy (AFM) and scanning tunnelling microscopy (STM), to change the substrate’s chemical or structural properties within the probe’s immediate vicinity [13]. For example, thermal scanning probe lithography (tSPL) is a maskless lithographic technique that employs a cantilever with a heated probe to pattern a material at the point of interaction with the surface. Patterning can occur through various mechanisms, including material removal, heat-induced physical or chemical conversion, or material addition by coating the tip in the material and depositing it onto the surface. The patterning outcome is influenced by factors such as tip shape and diameter, contact duration, force between the tip and the substrate, substrate thermal conductivity, and activation energy [145, 146]. Other SPL methods include dip-pen scanning probe lithography [147, 148], mechanical SPL [149, 150] and oxidation SPL [151, 152].

Overall, SPL techniques are cost-efficient, can be carried out in ambient conditions and substrates do not require further development [26]. The typical resolution of SPL varies between 4 and 10 nm, depending on the type of SPL [52]. Although SPL often has a low throughput, this can be improved by using multiple tips in parallel to improve the production speed [21]. However, each SPL technique has its own drawbacks. For example, in the case of tSPL, the achievable resolution diminishes with tip use, degrading over time. Moreover, the throughput of tSPL can be low due to either the mechanical movement of the probe or the reaction rate on the surface, however this can be improved by running multiple tips in parallel or by integrating a laser into the tSPL system [153].

SPL has recently been employed for replicating biological microenvironments. Tang et al. [154] demonstrated the application of tSPL in patterning cell culture substrates to mimic tissue microenvironments and their functionality. Following this, Liu et al. utilised tSPL to create replicas of bone tissues using a biocompatible polymer resist. This polymer enabled the growth and proliferation of stem cells, establishing a biomimetic system for studying and manipulating cell behaviour. tSPL was chosen over alternative lithographic techniques for its ability to efficiently pattern biocompatible materials at nanoscale resolution. Modifications to tSPL, involving the introduction of intelligent software and post-patterning procedures, along with the use of functional, biocompatible polymer resist proven to endure multiple cell culture cycles, reduced the cost and increased the throughput of surface production. This will enable the rapid prototyping of nanoscale topographies for biological studies [155].

The limitations of SPL in the biomedical industry depend on the specific SPL technique employed. Each technique has distinct material restrictions and resolution. For instance, dip-pen scanning probe lithography has limited control over depositing biomolecules on the substrate, while t-SPL cannot directly fabricate biomaterials due to the heat-induced denaturation of proteins. Furthermore, enhancing the low throughput by employing multiple probes increases the cost and complexity of the system, present challenges for the mass production of biomedical devices [52].

## Emerging lithographic techniques

### Evanescent near-field optical lithography

Evanescent near-field lithography (ENFOL) occurs within the near-field limit, where the separation between the wafer and the mask is held at a distance much smaller than the wavelength of the incident light, commonly 193 nm [68]. This causes the UV light passing through the photomask to form evanescent waves, which have high spatial frequencies [31] and thus gives a larger effective numerical aperture, which therefore can achieve resolutions down to 26 nm [156]. As evanescent waves decay exponentially, flexible photomasks are often employed to ensure the proximity of the photomask to the wafer at all points [68, 157]. ENFOL has the ability to produce nanostructures at a low cost, however it is not a widely used technique, which could be due to limitations such as exposure of unwanted areas of the substrate, the depth of field reducing as the structure size decreases and the stringent requirements for the mask [158, 159].

A schematic of ENFOL is shown in Fig. 1e [89]. The capability of ENFOL to generate cost-effective high aspect ratio structures holds promise for diverse applications including biochips in biomedical applications as well as nanoelectronics and nanophotonic crystal fabrication. For instance, Murukeshan et al. [160] used ENFOL to fabricate nano-dumbbells for frequency selective surfaces. Nevertheless, before it gains widespread acceptance in these industries certain challenges must be addressed. Maintaining a constant nanometer-scale working distance between presents an engineering challenge, particularly when considering any variations in the roughness of the substrate and the mask, with any deviations in this working distance across the substrate resulting in loss of resolution and fidelity. Overcoming these challenges is crucial for its successful industrial integration [161, 162]. However, given the existence of commercially available lithographic systems like EUVL and EBL, which already deliver high-resolution nanostructures cost-effectively for applications such as biosensors and cell studies, the time and cost involved in surmounting these engineering challenges could potentially present a less attractive proposition.

### Immersion lithography

**Fig. 2:**
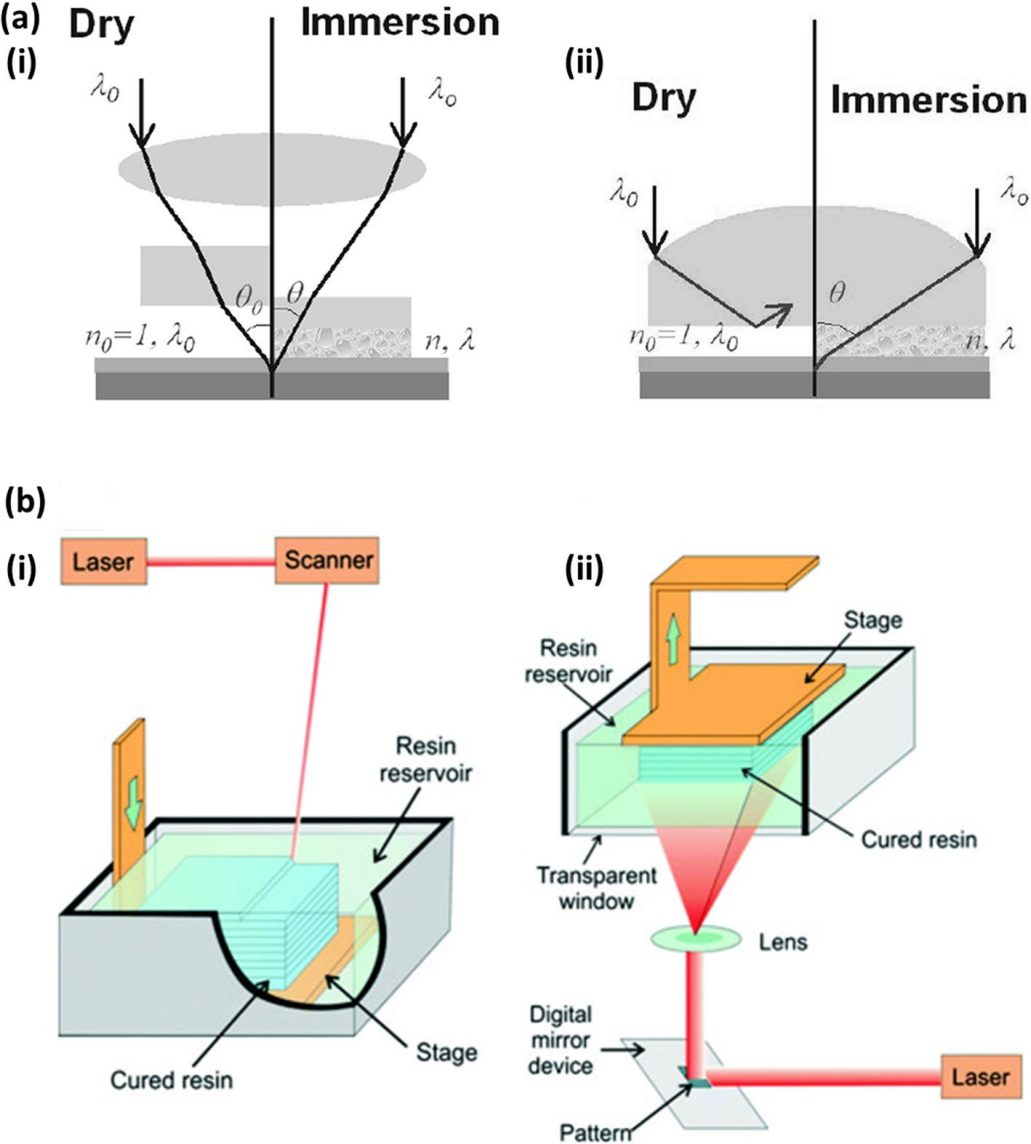
**a** Benefits of immersion lithography. **(i)** Increased depth-of-focus due to a smaller angle θ in the coupling medium. **(ii)** Increased resolution with higher numerical aperture optics by coupling light at larger incident angles. Image from [167]; reprinted with permission from [166]. **b** Two typical implementations of stereolithography for rapid prototyping of ceramics. **(i)** *Top-down* system with scanning laser on top and **(ii)** *bottom-up* systems with digital light projection. Image from [173]

Immersion lithography (IL) was developed with the aim of improving the resolution of PL by placing a transparent liquid, with a refractive index greater than air, in between the final piece of optical apparatus and the photoresist [71]. The coupling between the light and the fluid increases the critical angle and therefore enables the application of shorter wavelengths [163]. The effective radiation wavelength ${\uplambda }_{\mathrm{eff}}$ in a fluid with refractive index ${\mathrm{n}}_{\mathrm{f}}$ is given by:

$${\uplambda }_{\mathrm{eff}}=\frac{{\uplambda }_{0}}{{\mathrm{n}}_{\mathrm{f}}}$$

where ${\uplambda }_{0}$ is the wavelength in a vacuum, leading to the effective wavelength being reduced [164]. IL can be combined with DUVL, utilising wavelengths of 193 nm to generate resolutions of up to 38 nm [165]. These advantages of immersion lithography over PL are overviewed in Fig. 2a [166].

However, challenges arise concerning the fluid medium. It must meet several requirements including having a high refractive index to lower the effective wavelength and being highly transparent. Furthermore, since the fluid can enhance both heating effects and the likelihood of radiation damage, properties such as low viscosity, low coefficients of absorption and thermo-optics, high specific heat capacity and high thermal conductivity are all advantageous and reduce the likelihood of spherical aberrations. Water can meet these requirements, although contaminants such as biomolecules, compounds, particles, and dissolved gases must be removed to prevent unwanted absorptions. The interactions between the photoresist and water, and the effect these interactions have on the emergence of defects and the accuracy of the pattern formation, must be well understood [167].

Gil et al. [168] first manufactured microprocessors with immersion lithography in 2005 and showed promise for the application of immersion lithography in the semiconductor industry. Furthermore, Hu et al. used immersion lithography to produce a colour display meta-surface with high resolution and enhanced efficiency compared to displays created with EBL, offering an alternative production method for the mass production of flat optic devices for applications such as biosensing and high resolution biomedical imaging [169, 170].

Nonetheless, the application of immersion lithography in the biomedical sector may face limitations. In biomedical settings, where manufacturing environments must adhere to stringent cleanliness and sterility standards, the incorporation of a liquid immersion medium presents challenges. Careful selection of the liquid and photoresist used in immersion lithography is imperative to prevent the introduction of contaminants or any adverse effects on biological materials or delicate medical devices [171].

### Stereolithography

3D printing (also known as additive manufacturing) techniques have been revolutionising prototype design, allowing emerging start-up companies to quickly design and build devices as well as similarly, research laboratories with custom experimental set-ups to easily produce bespoke components. A prominent 3D printing approach is stereolithography (STL), which takes advantage of UV laser beams interacting with photopolymer resin, depositing and curing it layer-by-layer to fabricate a structure. The incorporation of photoinitiators and UV absorbers within the resin increases the possible depth of polymerisation [172]. Two typical arrangements for patterning via STL are overviewed in Fig. 2b [173].

STL enables the structuring of viscous resins and can produce complex structures over a large area [172, 174]. Through advances in STL, including the micro-STL and projection micro-STL, resolution of 0.6–10 µm have been achieved [175, 176]. However, STL is limited to photopolymers and therefore the production of new photocurable media has been explored [177]. It must fulfil many requirements, including being nontoxic, soluble before curing and insoluble after curing, fast to cure, able to adhere to the previously deposited surface to enable layer-by-layer formation, high absorption coefficient and low viscosity [178]. Furthermore, it is an expensive process which must be efficiently ventilated to remove the fumes produced and the resins must be carefully contained [179]. Print angle of the emerging design and consequently, the support structures must be carefully considered for optimal fabrication. Printed parts must also be washed and cured, extending the processing time. In contrast, such steps are unnecessary in fused deposition modelling (FDM) 3D printing, which uses a heated thermoplastic filament.

Zhang et al. integrated wood flour into resins before manufacturing composites via STL. The addition of wood flour improved the tensile strength and Young’s modulus of the composite whilst exhibiting stress-whitening behaviour, enabling the observation of stress within the material [180]. Furthermore, STL has been used in the medical industry, with Ullah et al. [181] exploring the application of STL with calcium phosphate nanoparticles to create bone scaffolds to aid in their repair after injury. Moreover, Robles-Martinez et al. employed STL to generate polypills which have a multi-layered structure. This enables the patient to ingest multiple drugs within one capsule and therefore improves the probability that a patient will take all of their required daily medications. STL offered a method of efficiently fabricating high resolution polypills without the risk of thermal degradation [182].

Obstacles associated with employing STL in the biomedical sector involve the choice of biocompatible resins, particularly given the lack of standardisation regarding the amount and quality of information provided with these materials [183]. Additionally, bio-related components often require sterilisation, which could potentially damage their properties, and as of now, there are no existing standards or guidelines for sterilizing 3D-printed materials [184]. The broader utilisation of STL in biomedical applications awaits further standardisation and regulations to address these challenges.

### Two-photon lithography

**Fig. 3:**
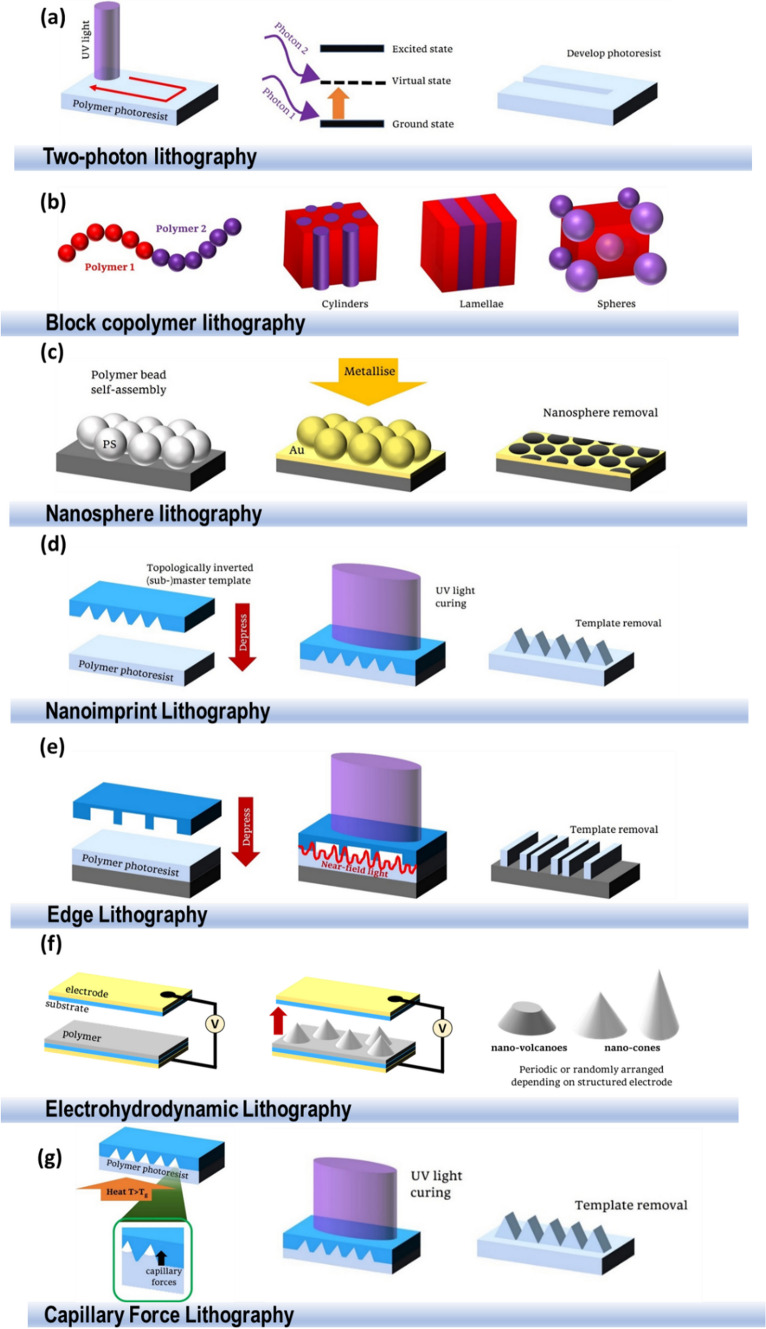
**a.** Two-photon lithography. Two UV photons react with the photopolymer to produce a pattern. **b** Block copolymer lithography. A block copolymer and the various structures which can be produced by altering the ratio of monomers. **c** Nanosphere lithography. Nanobeads are deposited on a substrate and metallised. The nanospheres are then removed to produce the patterned substrate. **d** UV nanoimprint lithography. The photopolymer fills a stamp and is cured with UV light and the stamp is then removed to produce the final structure. **e** Phase-shift edge lithography. The photopolymer fills a stamp, and the constructive and destructive interference of light cures areas of the photoresist. The stamp is removed to produce the final pattern. **f** Electrohydrodynamic lithography. A voltage applied across parallel plates induces instabilities in a polymer film with the possibility of producing various nano geometries. **g** Capillary force lithography. The polymer is heated and fills the stamp through capillary forces. The polymer is then cured, and the stamp is removed

Two-photon lithography (TPL) is a novel fabrication technique which, like PL, utilises resist and UV light to create patterns (Fig. 3a**)**. The resist undergoes a two-photon polymerisation process, whereby the first photon is fired at the photoresist and is absorbed by a photo-initiator molecule, exciting it to an intermediary virtual state, which returns to its ground state within femtoseconds. Before this decay can occur, a second photon interacts with the photoresist to further excite the molecule, leading to the polymerisation and hardening of the resist. The pattern is defined digitally, and the UV laser beam is moved according to this pattern to reproduce the design upon the photoresist [185, 186].

This technique can generate three-dimensional patterns by exposing the UV laser to areas known as voxels and changing the laser intensity adjusts the dimensions of the pattern. The use of two photons results in the intensity function being squared such that smaller dimensions can be generated [185], leading to resolutions down to 150 nm. Metallic materials cannot be used with this technique however due to the need for a photoresist [187], and there are issues with the time required to print large structures and the amount of data storage required for the digitally-defined pattern [185].

TPL has been used in several medical applications owing to its high resolution and capacity to create three-dimensional structures. Marino et al. used TPL to create microtubes to grow cells which mimic brain capillaries, enabling a model of the blood–brain barrier to be developed to simulate the passage of drugs through the blood–brain barrier before testing in human trials [188]. Larramendy et al. [189] also used TPL to produce a scaffold for cell growth which could be further developed into artificial organs or used for drug screening. Recently, Limongi et al. [190] employed TPL to produce vascular grafts to improve blood flow to an organ affected by a diseased artery.

TPL encounters challenges in the biomedical industry due to the restricted availability of biocompatible photosensitive polymers [191]. Additionally, the cubic relationship between resolution and fabrication time diminishes the throughput of high-resolution tissue engineering scaffolds of sizes relevant to physiological requirements. To achieve widespread commercial use, extensive research is needed to devise methods to reduce fabrication times such as producing a master structure with TPL and creating replicas via soft lithography, producing shell structures, or by utilising multiple laser beams [192].

### Block copolymer lithography

Block copolymers are polymers that consist of several different monomers. For example, di-block copolymers are generated from two non-identical monomers which are covalently bonded in one region. An end block which can react with both monomers may be required to connect them. As these monomers are often immiscible, they will attempt to phase separate but will be prevented from completely doing so by the covalent bond linking them together. Annealing the block copolymers generates the energy required for the monomers to become motile and separate into organised microdomains, as demonstrated in Fig. 3b. The final structure is dependent on the volume fractions of the monomers, the different possible phases include spheres, cylinders, ordered bi-continuous double diamond and lamellae [193]. In block copolymer lithography (BCL), the block copolymer can be spin-coated onto a substrate to produce a pattern and using an etch a replica can be generated on other varying substrates [194].

BCL is a simple and inexpensive technique which can generate precise patterns over large areas [195]. With the use of topographical guiding patterns to direct the self-assembly of the block copolymers, sub-10 nm resolutions have been achieved [196, 197]. However, the structures may not arrange in the optimal configuration for a specific application as one of the monomers may be more likely to wet the substrate [198] and the periodicity of the microdomains can limit patterning possibilities [193, 199].

BCL was used by Kim et al., where the authors spin-coated a 120 nm layer of polystyrene block poly(methylmethacrylate) (PS-b-PMMA) onto a silicon wafer to produce a hollow co-block polymer nanohole array. The structures served to suspend 2D materials, improving the material transport properties by minimising phonon scattering for graphene field-effect transistor applications. BCL demonstrated advantages such as its simplicity, low cost and scalability over conventional top-down methods [200]. Elsewhere, Banbury et al. explored four different types of nanostructures produced with BCL for plasmonic sensing applications via SERS. Gold-coated gyroidal and cylindrical structures were fabricated, focusing on optimisation of fabrication parameters such as the block copolymer fractions. The authors found that maximum SERS enhancement factors were recorded for free-standing gyroids interrogated at 633 nm and 785 nm with a benzenethiol analyte [201]. Elsewhere, the capability to produce high-resolution structures across large areas has resulted in the adoption of BCL in the biomedical industry. Jeong et al. employed BCL to create a silicon nanomesh for the electrical detection of biomolecules [202] and Shin et al. utilised BCL to fabricate hexagonal arrays of noble metal nanoparticles for plasmonic biosensors [203].

BCL in the medical sector is limited to the use of biocompatible block copolymers. Comprehensive research and understanding of the interactions between these polymers, along with the optimisation of conditions such as temperature, are essential to generate reproducible high-resolution patterns with minimal defects for biomedical applications [204, 205].

### Nanosphere lithography

Nanosphere lithography (NSL) is a fabrication method which creates single layers of periodic nanoparticles [206] (Fig. 3c). A flat substrate is first chemically treated to increase its hydrophilic nature and is covered in a liquid containing suspended monodisperse colloidal nanospheres such as polystyrene. Once dry, a monolayer of colloids is formed in a hexagonal close-packed topography, known as a colloidal crystal mask (CCM). A material can then be deposited onto the mask and interacts with the substrate in areas not obstructed by the colloids. The mask can then be removed using techniques such as stripping or sonication with a solvent, producing a pattern of nanodots on the substrate. Annealing this final product can be necessary to crystalize the material or change its crystallographic phase [207].

The production of CCMs can be achieved with several techniques such as:

- *Langmuir–Blodg*et*t (LB) coating* the colloidal suspension is deposited on the surface of the substrate, with either the substrate or the suspension being hydrophobic. Hydrophobicity can be induced via the use of surfactants or changing the nanosphere surface. The hydrophobicity causes the nanospheres to separate and spread across the substrate, creating a 2D crystal film. A barrier is then applied to the film, compressing it to form compact nanosphere lattices [206]. LB coatings generate highly ordered and uniform monolayers of nanospheres [208] However, the absence of bonds between the nanospheres and the induced charge build-up generates repulsive forces between nanospheres, and unless the substrate is completely flat the preservation of the nanosphere lattice structure is challenging [209].
- *Solvent evaporation* droplets of the colloidal nanosphere suspension are placed onto the substrate. The solvent then evaporates and the combination of the convective transport of the nanospheres and the attractive capillary forces due to the meniscus between the nanospheres, which can be controlled by the solvent evaporation rate, causes the self-assembly of nanospheres [210, 211]. Solvent evaporation is a simple and inexpensive technique, although the orientation and symmetry of the crystal film formed cannot be controlled [212].
- *Dip-coating* the substrate is submerged and then removed from the colloidal suspension. Like the self-assembly via solvent evaporation method, the solvent evaporation rate controls the order of the nanospheres. In dip coating, the evaporation rate can be managed by a stepper motor, which raises the substrate slowly from the colloidal suspension [213]. Unlike methods such as LB, the nanospheres do not need to be treated with chemicals, reducing the process complexity. On the other hand, dip-coating is not efficient as the nanospheres attach to the surface of the container the suspension is held within, decreasing the concentration of colloids that attach to the substrate. The concentration of nanospheres therefore must be increased which in turn increases the cost of the process [214].
- *Spin coating* the colloidal suspension is deposited on the substrate and revolved, increasing the solvent evaporation rate as it is dependent on spin velocity and acceleration. It also depends on the size and concentration of the colloidal suspension, wettability of the substrate, pressure and humidity. Spin coating is a common laboratory technique since it is highly efficient and the factors which influence the solvent evaporation rate can be controlled, although as these factors are dependent on each other some experimentation is required to determine the optimal conditions [215].
- *Electrophor*et*ic deposition* an electric field is applied to the colloidal suspension by placing it in between two electrodes. The nanospheres proceed towards one of the electrodes, where self-assembly occurs. This is possible with both AC and DC electric fields, although AC fields do not give rise to water electrolysis at high field strengths and can be used with a wider range of particles and substrates. As the substrate must also be an electrode, it must consist of a conductive material. Despite the high electric fields, the nanospheres can remain in Brownian motion whilst interacting with the electrode, to prevent this a layer of surfactant can be adsorbed to the nanosphere surface [216, 217].

NSL is a relatively inexpensive lithographic method able to generate versatile periodic structures over large areas, with control over factors such as the dimensions and material of the patterns [218]. Furthermore, it is possible to fabricate nanoscale structures with micrometre nanospheres, as the fabricated structures tend to have dimensions approximately a fifth of the dimension of the nanospheres. Although resolutions of 1 nm have been demonstrated [219], NSL has restricted pattern geometries, as the dimensions of the structures and their separations tend to be inter-dependent. There is also difficulty in controlling the crystal domain size defects and dislocations [220].

Gao et al. used NSL to design palladium-silicon nanomeshes for hydrogen detection. The authors achieved this by first creating a layer of polystyrene spheres on a substrate, depositing a layer of chromium onto the substrate and then removing the spheres leaving a nanomesh of chromium. This nanomesh was then used as a hard mask for reactive ion etching to replicate the nanopattern onto a silicon substrate, with dimensions of 20 µm. This method produced a sensor with improved sensitivity and selectivity and at a much lower cost than sensors previously produced via conventional techniques such as EBL [221]. Similarly, Brinkert et al. employed shadow nanosphere lithography for hydrogen generation. Rhodium nanostructures were deposited onto indium phosphide substrates to produce 3D nanostructures for high-activity photodiode—electrocatalyst networks, able to generate hydrogen whilst in microgravity. These platforms could offer a solution to fuel storage limitations during space explorations by being able to continually generate the fuel required [222]. Recently, Jin et al. implemented NSL to produce SERS substrates. A combination of polystyrene NSL and wet etching generated periodic, bioinspired silicon pyramid nanostructures with antireflective capabilities. Silver nanobowls were produced on these pyramids to form SERS substrates with the potential to detect trace concentrations of dyes [223]. Lei et al. also used polystyrene nanospheres to design gallium nitride (GaN) nanorods for indium gallium nitride/gallium nitride (InGaN/GaN) light-emitting diodes (LEDs). NSL enabled the production of low-cost nanostructures on large, uniform substrates to generate InGaN/GaN LEDs, with an improved light extraction efficiency, for applications in areas such as optics and underwater communications [224]. Kim et al. [225] developed surfaces featuring nanowell arrays to facilitate neuronal cell growth, while Purwidyantri et al. [226] created gold nanohole arrays to enhance the electrochemical performance of DNA biosensors.

Another device found to be beneficial to healthcare is the vertical field effect transistor (VTFET), the development of which has enabled an increased number of transistors to be positioned within a 2D area in comparison to planar FET, producing devices with higher current densities, on/off ratios and frequencies and lower voltage requirements [227, 228]. Biosensors based on FET devices have exhibited high specificities and sensitivities, rapid result generation and can be easily integrated with further systems to mass-produce biosensors at a low cost [229]. Applications for FET-based biosensors include the detection of cancers [230, 231], COVID-19 [232, 233], myocardial injury [234], monitoring of blood glucose levels [235], radiation dosage [236] and skin temperature [237]. Emerging lithographic techniques have the potential to improve the fabrication of VTFETs, as demonstrated by Chang et al. where the authors employed surfactant-assisted NSL to generate the perforated source electrode for their vertical organic FET (VOFET), displaying an improved mobility with on/off ratio [238].

Unlike conventional methods like EBL and FIBL, the constrained pattern geometries produced by NSL restrict its versatility in the biomedical industry. Additionally, NSL faces challenges in scalability since generating large areas of the nanosphere mask proves difficult, impeding its application in the mass production of medical devices. Research aimed at developing improved photomasks and exploring self-masking techniques may provide potential solutions to overcome this issue [239].

### Nanoimprint lithography

Nanoimprint lithography (NIL) involves spin-coating a polymer resist onto a substrate and pressing a stamp onto it; the polymer then fills the stamp, replicating the stamp’s pattern in the polymer [240]. NIL stamps thus, provide rapid, high-resolution nanopatterning with high-throughput and low cost, tackling industrial needs [241]. Recently, Liu et al. [242] highlighted the ease of production of structures via NIL as useful in augmented reality glasses, vehicle lighting and 3D displays. Whilst the NIL stamp can be hard or soft, for harder silicon-based templates, there are difficulties which occur when eliminating defects and removing the stamp from the substrate due to deposition at its edges, both of which can cause damage to the stamp. Soft stamps, such as the polydimethylsiloxane (PDMS) stamps used in soft lithography, are more flexible and therefore are less susceptible to damage in the presence of unwanted particulates. Their flexibility also ensures the contact area between the stamp and the substrate is maximised. However, soft PDMS stamps cannot produce structures with high aspect ratios due to a low Young’s modulus causing collapse, unless harder PDMS is used [243, 244]. Furthermore, the ability of PDMS to absorb organic solvents can induce swelling in the stamp, reducing the quality of the fabrication [245].

There are various types of NIL. For example, hot-embossing NIL (HE-NIL) involves pressing the stamp down onto a thermoplastic polymer and heating it above the glass transition temperature *T*_g_, such that the polymer changes into a more flexible state and fills the stamp. Once cooled, the stamp is removed, leaving a pattern on the substrate. Although HE-NIL is often preferred in industry as it is a much simpler technique and a larger range of polymers can be patterned [246], the thermal expansion coefficients of the substrate and the stamp vary and therefore can cause misalignment errors [247]. Furthermore, it is challenging to achieve high aspect ratio fabrications, due to the large polymer-stamp contact area and the inability to control the surface energy of the stamp material [248]. This can be combatted by performing sequential HE-NIL whereby, once the first stamp has been removed, a different specifically orientated stamp is placed onto the patterned polymer once it has cooled below *T*_g_, producing a secondary fabrication [249]. Another type of NIL is ultraviolet NIL (UV-NIL), which utilises a liquid photopolymer to fill the stamp and is solidified with UV light before the stamp is removed (Fig. 3d) [250]. The low viscosity of the curable polymers means that only low pressure must be exerted on the stamp and the polymer can fill high aspect features in the stamp, resulting in accurate replicas of the stamp in the polymer. The short polymer curing times enables a high throughput and, unlike HE-NIL, UV-NIL can be carried out at room temperature. However, UV-NIL is limited to UV-curable polymers and the application of UV radiation must be carefully controlled, too little irradiation results in incomplete curing such that the structures may be weak and deform, whereas excess irradiation can cause the resist to shrink and become brittle, leading to fracturing when the stamp is removed [251]. Although UV-NIL can be carried out in ambient conditions, low imprint pressures can lead to bubble defects, to reduce the presence of these defects UV-NIL can be carried out under vacuum, the imprint pressure can be increased, or the bubbles can be removed via gas condensation with pentafluoropropane [252].

One example of UV-NIL is Jet and Flash Imprint Lithography (J-FIL), which can produce features with dimensions below 20 nm. It involves dispensing drops of resist through an inkjet onto a substrate, coated with a thin adhesion layer that promotes wetting, and the substrate position is adjusted such that the resist drops align accurately with the template. Using air pressure, a bow of approximately 10–15 µm is induced in the stamp to ensure sufficient contact with the resist drops, and the template bow is then relaxed using vertically moving actuators. Prior to UV curing, nanoscale alignment or overlay must be ensured, before finally separating the template without shearing any nanoscale features. J-FIL is the primary non-optical lithographic method that enables the production of advanced integrated circuits for industry [253].

NIL is a precise and inexpensive technique which has a high throughput and can produce nanoscale features over large areas [218], resolutions below 3 nm have previously been achieved. The main issues arising from NIL are associated with the interactions between the various surfaces. If the interaction between the stamp and the polymer is too strong, it becomes difficult to separate them and defects can be generated in the polymer remaining on the substrate. On the other hand, if the interaction between the polymer and the substrate is too weak the pattern from the stamp is not accurately transferred onto the substrate. To minimise these effects the surface chemistry of these surfaces has been explored and modified to produce the required result [254]. An alternative is to use an anti-stick coating to prevent the adhesion of the stamp to the polymer [255]. An et al. pointed out that residues will inevitably accrue in the submaster template mold, which on one hand could be preventing damage to the template stamp, and on the other, affecting nanostructure morphology and thus, reducing the fidelity [256].

Zhou et al. produced plasmonic nanocave arrays for integration into a multichannel microfluidic chip for the detection of tumour biomarker carcinoembryonic antigen (CEA). The authors recorded a maximum refractive index sensitivity of 490 nm/refractive index (RI) unit in the visible range, which compares favourably to other NIL-based plasmonic sensors. Consequently, a detection limit of 5 ng/mL is observed, which is below the cancer diagnosis threshold of 20 ng/mL. The chip is functionalised with the anti-CEA antibody and thus offers selective detection. No RI change was seen for alternative biomarkers ferritin (Fer) and alpha 1 fetoprotein (AFP). Moreover, the reusability of the device was demonstrated via the application of 0.5 mol/L hydrochloric acid with minimal detriment to performance [257]. Recently, Panneerselvam et al. has summarised the potential of combining microfluidic systems with plasmonic enhancement nanostructures that could also benefit from cheap high-throughput techniques like NIL [258]. Müsse et al. have used NIL to fabricate a wearable microfluidic glucose current sensor for sweat analysis, which, like blood and tear samples, and saliva in certain health conditions, are often available in small (mL) quantities. Over a pH range of 4.5–8.0, glucose oxidase was monitored and a linear range of 0.025 mM to 2 mM determined [259]. Chen et al. proposed microfluidic device fabrication via UV-NIL, conferring benefits of not only low-cost and flexibility but crucially scalability, a perceived bottleneck to commercial adoption. Channels of lateral and depth sizing of 1 mm and 55 μm were generated. The authors detected salmonella at a colony-forming unit /ml of 10^5^ at a statistical significance *P* < 0.01 [260]. Bacterial detection has implications not only for healthcare but in the overlapping area of food safety, where microfluidics is also an emerging research domain.

Meanwhile, Li et al. employed a PDMS stamp in a NIL process to produce an ultrasonic micro-ring resonator structure which was used in a cranial window device for longitudinal monitoring of cerebral vasculature, where haemodynamic analysis may be useful in a range of conditions, including traumatic brain injury and Parkinson’s disease. The authors monitored murine cortical activity at an axial resolution of approximately 4 μm for 28 days, noting a minor decline in resonator performance. Losses were minimised by selection of low-loss polystyrene, and prior treatment of the silicon master template to reduce surface roughness. The soft NIL approach permits an inexpensive disposable device that may be easily fabricated in a fume cupboard with a hotplate [261].

Furthermore, Carthew et al. studied the effect on cell outcomes via changes to the underlying microtopography with a view to applications in stem cell culturing and smart implant materials. Using UV-NIL to fabricate nanopillars of varying sizes and periods, and mesenchymal stem cells as a model, the authors reported alterations to the nuclear architecture depending on specific substrate topology. For instance, *β*-actin, compared to a flat surface control, is reduced by a factor of 2 on the 1 μm × 1 μm × 5 μm array, and further to a factor of 3 on an array with dimensions 10 μm × 10 μm × 5 μm (L × W × H). Moreover, nuclei morphological changes were noted where ‘indentations’ and ‘holes’ are apparent, contingent on the underlying structure [262].

NIL encounters challenges that hinder its application in specific areas of the biomedical industry. For instance, the direct printing of proteins onto substrates for cell biology studies proves challenging due to the potential denaturation of proteins caused by the UV light in UV-NIL and the high temperatures in HE-NIL. To address this, patterns can be created through NIL for subsequent protein immobilisation [77]. Moreover, the widespread use of NIL for manufacturing devices such as microfluidic chips and applications in cell studies is restricted by the lack of uniformity and reproducibility. This limitation stems from stamp-substrate interactions, as well as the application of pressure and temperature to the stamp during imprinting processes, leading to pattern warping and stamp deformation. Research aimed at reducing defects in NIL, employing methods such as introducing local mold deformation along the block boundary and applying biocompatible anti-stick coatings, is crucial for establishing NIL within the biomedical industry [263, 264].

### Edge lithography

Edge lithography (EL) (Fig. 3e) encompasses a group of techniques that uses topographic edges to pattern arrays, which are fabricated in parallel and have dimensions below 100 nm, in a range of different materials [53]. It involves the selective deposition or removal of materials on the edges, after or in conjunction with other conventional techniques such as PL [265]. One type of EL is topography-directed pattern transfer, an example being phase-shift edge lithography. This uses the vertical edges of a topographic transparent mask and when a collimated beam of light is incident on the mask it induces a phase change, producing small areas of constructive and destructive interference on the photoresist. This can improve the resolution such that 30 nm structures can be generated. Another category of EL is cutting or cleaving of an edge to reveal an edge with nanoscale features, for example by continually exposing the target material to a rough surface to produce nanostructures on its edge [53]. EL can be used as an intermediate step in the production of stamps for fabrication methods such as HE-NIL. It increases the nanofabrication efficiency [266] and improves the mechanical strength and shape of the fabricated nanopatterns [265] and reduces the overall cost. EL can improve the resolution of other lithographic techniques, although it only covers relatively small areas [53].

Other variations of EL include graphene edge lithography, on-edge lithography, etching at edge defects in self-assembled monolayers or undercutting effects [53, 266, 267]. Chen et al. have used edge effects in NIL to tune the dimensions of wet-etched volcano-shaped sapphire nanostructures on a substrate for light-emitting diode (LED) applications. The authors vary the time for a reactive ion etch to alter ring widths by 45 nm. Reduced threading dislocation results in an increased quantum efficiency of 24% over conventional patterned sapphire substrate methods [268]. Meanwhile, Solak et al. [269] introduced ‘displacement Talbot lithography’ based on the Talbot effect, where incident plane EM waves produce images at repeated intervals when a periodic diffractive grating is illuminated, to produce nanostructures without the need for projection optics. More recently, the same authors have combined this approach with phase-shift edge lithography to form desired nanostructure topographies independent of mask-surface separation [270]. Additional applications which benefit from the advantages of EL include the production of flexible electrodes, piezoelectric devices and omniphobic surfaces [271].

In the biomedical field, Xie et al. employed edge lithography to fabricate high-aspect ratio nanochannels, applicable to micro-nanofluidic systems like biosensors. This method was preferred over conventional techniques such as EBL and FIBL due to its cost-effectiveness and higher throughput [272]. Moreover, Vafai et al. applied evaporative edge lithography to design multilayer liposomal microarrays tailored for a cell-based migration assay. Understanding cellular migrations holds key insights into phenomena like wound healing, cancer metastasis, and angiogenesis, bearing implications for high-throughput screening and innovative drug discovery. Diverging from traditional migration assays, this approach facilitates the screening of various small molecule compounds and dosages on the same surface without compartmentalisation. It exhibits promising scalability for high-throughput screening of small lipophilic molecules, and the miniaturisation process opens avenues for producing portable small molecule libraries on a chip [273]. Nevertheless, to fully integrate EL into the biomedical industry, further investigations are needed to explore the range of materials amenable to patterning, enhance pattern resolution, expand the patterning area, and engineer porous structures or intricate hierarchical architectures essential for applications like biosensors [271].

### Electrohydrodynamic lithography (EHL)

Electrohydrodynamic Lithography (EHL) is a one-step patterning method in which an electric field is manipulated to destabilise thin-film polymers, as shown in Fig. 3f [274]. EHL involves spin-coating a thin polymer film onto the bottom electrode, producing a homogeneous film. The application of polystyrene nanoparticles in the corners of the electrode allows a nanoscale air gap to be maintained between the bottom and an upper electrode. The polymer is heated above its glass transition temperature such that it transitions from behaving like a solid to a viscous liquid. Then applying a voltage across the electrodes produces a homogeneous electric field of the order 10^8^ V/m. The electric field gives rise to the accumulation of displacement charges at the polymer-air interface, which are attracted to the top electrode and creates an effective surface charge density. The interaction between the displacement charges and the electric field produces an interfacial electrostatic pressure which overcomes the stabilising surface tension. The coupling of the electrostatic pressure to the capillary waves which are present due to the Brownian motion of the molecules in the film, stemming from the surface of the polymer film not being completely flat, amplifies or damps the instabilities. The amplifications occur with a characteristic wavelength whilst the polymer flows such that it is redistributed laterally, there is a decrease in the amount of polymer in areas in which damping occurs and this flows to areas in which amplification occurs, leading to the build-up of polymer in these regions. The polymer grows vertically by draining the liquid bridges coupling it to the bottom electrode until it meets the top electrode. This produces hexagonally symmetric spaced polymer pillars, which is the most energetically favourable state as it reduces the repulsion between the extrema of the polarised fluctuations. The inter-pillar distance is dependent on the surface tension of the polymer and the electric field strength, whilst the pillar diameter depends on the original film thickness when it is spin-coated onto the substrate and the air gap between the two electrodes [275].

Using a planar electrode and a homogeneous electric field, the polymer pillars and their inter-pillar spacing are of the order of micrometres. To reduce these dimensions, a topographic structure can be imposed onto the originally planar top electrode with another lithographic technique such as EBL. When an external electric field is applied, the topographically structured electrode acts as an equipotential surface. By varying the spacing between the lithographic structures on the upper electrode, $\mathrm{d}$, a laterally heterogeneous field is generated, and the instabilities are directed towards the areas with the highest electric field, as the electric field is inversely proportional to $d$, the instabilities and the liquid polymer are directed towards the lithographic structures to produce a positive replica of the topography. Using this technique, lateral length scales of the order of nanometres can be generated [276].

Overall, EHL is a simple, low-cost and versatile lithographic method able to produce high-resolution structures [277], with resolutions below 50 nm previously being achieved [278]. Furthermore, it has been previously demonstrated that other materials such as carbon nanotubes can be combined into the polymer pillars, which can enhance or change their functionality and characteristics [277, 279, 280]. The generated pillars may not be uniform, as there is an inherent degree of misalignment between the electrodes during the patterning process and various aspect ratios of the formed structures can be observed on the same substrate laterally. The larger the angle of misalignment, the larger the inhomogeneity of the pillars. The final step of EHL, removing the top electrode, can also damage the pillars [281]. Moreover, EHL requires carefully choosing the optimal fabrication time, since if the time is too short the pillars will not fully form, whereas if the time is too long, the pillars tend to coalesce, reducing the accuracy of the final pattern. The challenge of the long fabrication time has been overcome by reducing the EHL patterning time from hours to seconds by utilising low-viscosity polymers [282].

EHL can be used for a variety of applications, including for instance, to manufacture substrates for SERS sensing applications, [283] where Mahajan et al. gold-coated EHL pillar arrays to produce low-cost SERS substrates [276] and Goldberg Oppenheimer et al. investigated the use of multilayer thin films to produce hierarchical electrohydrodynamic structures, which are then coated with a nano-gold layer to generate advanced SERS-active devices [284]. Busà et al. investigated producing EHL structures for tuneable superhydrophobic surfaces by manufacturing carbon nanotubes (CNTs) on the top electrodes. The CNT-patterned electrodes are highly durable, and their dimensions and layout are easily adjustable such that the structures produced via EHL can be generated over large areas at a relatively low cost with controllable hydrophobic properties [279]. Another application of EHL structures explored by Ding et al. is organic thin-film transistors. The authors found that producing these transistors with EHL was simpler and more cost-effective than current methods such as PL and EBL, and has greater potential for applications such as flexible wearable devices [285]. The ability to pattern the biocompatible polymer polycaprolactone, exemplified by Goldberg Oppenheimer et al., opens avenues for applying these flexible devices to the biomedical industry, including implantable medical devices [282, 286]. However, for widespread production and reproducibility in applications such as wearable biosensors, further research is necessary to enhance production techniques to improve the throughput, maintain a consistent nanometer-scale distance between electrodes for uniform structures to ensure reproducibility and explore the patterning of additional biocompatible polymers, thereby expanding its application possibilities.

### Capillary force lithography

Capillary force lithography (CFL) is viewed as a combination of NIL and soft lithography (Fig. 3g). Like NIL, CFL involves the structuring of a liquid polymer and uses an elastomeric mould such as in soft lithography. A polymer layer is spin-coated onto a substrate and the mould is placed on top of the polymer. The polymer is then heated above its glass transition temperature, *T*_g_, and capillary forces cause the polymer to rise and fill the gaps in the stamp. The polymer is cooled to below *T*_g_, leaving a pattern on the surface of the substrate, which is a negative replica of the original stamp, which is then removed. Although CFL relies on capillary action, it does not suffer from the low polymer filling rates, instead the rate is proportional to the stamp size. CFL is a versatile technique which can manufacture precise features. However, the aspect ratio must be carefully chosen. If the aspect ratio is too high the structures may collapse, whereas if the aspect ratio is too low the structure may not have the required stability. This limits CFL to low-density patterns with feature dimensions greater than 100 nm, unless other techniques are used to combat this *e.g.,* pressure, vacuum or solvent-assisted CFL [287].

Zou et al. used CFL to design linear arrays of palladium cubes. When hydrogen interacts with palladium cubes it undergoes dissociative absorption and changes the cube’s properties such as its electrical, optical and morphological characteristics. These arrays are therefore able to detect the presence of hydrogen gas, which is essential when considering the production of hydrogen for renewable energies. Conventional techniques for producing miniature sensors, such as electron-beam and ion-beam lithography, have a low throughput and high cost, whereas CFL is a high throughput technique and can easily produce large nanoarray structures with less expense [288]. Tan et al. also manufactured a sensor system with CFL, creating superhydrophobic SERS substrates able to detect ultra-trace elements for environmental and forensic applications. CFL enabled the production of box-like micropatterns on the surface of a polymer, before adding silver nanocubes and a final coating of silver and perfluorodecanethiol, to generate a high throughput of consistent, stable, low-cost SERS substrates [289].

In the biomedical industry, CFL has been employed by Tsui et al. to produce bioinspired cardiac scaffolds to grow human cardiac tissue for applications such as in vivo therapies and in vitro disease replication. Unlike methods such as electrospinning, CFL can produce nanostructures with high reproducibility and fidelity whilst being performed under ambient conditions [290]. Moreover, Hyun employed CFL to pattern a thin film of poly(ε-caprolactone) (PCL) into an array of disks and subsequent annealing yielded spherical particles. CFL facilitated the creation of isolated PCL disks, allowing them to produce spherical beads through thermal annealing. It also provided precise control over the resulting beads, with their diameter and thickness contingent on the diameters of the disks and the thickness of the PCL film, respectively. These beads exhibited promise as controlled drug delivery systems [291].

The incorporation of CFL into the biomedical industry requires further investigation. Issues such as interactions between the polymer and the stamp, as well as poor wetting of the stamp, may compromise the reproducibility of structures. While this can be addressed through slow evaporation or by reducing the patterning area, these solutions are suboptimal for the mass production of biomedical devices [292]. Additionally, similar to NIL, challenges may arise when integrating biocomponents, such as proteins, into the structure, as high temperatures could denature the proteins. Potential solutions include immobilizing proteins on the surface after structure fabrication or exploring CFL patterning at lower temperatures [287].

### Near-field electrospinning-assisted lithography

**Fig. 4:**
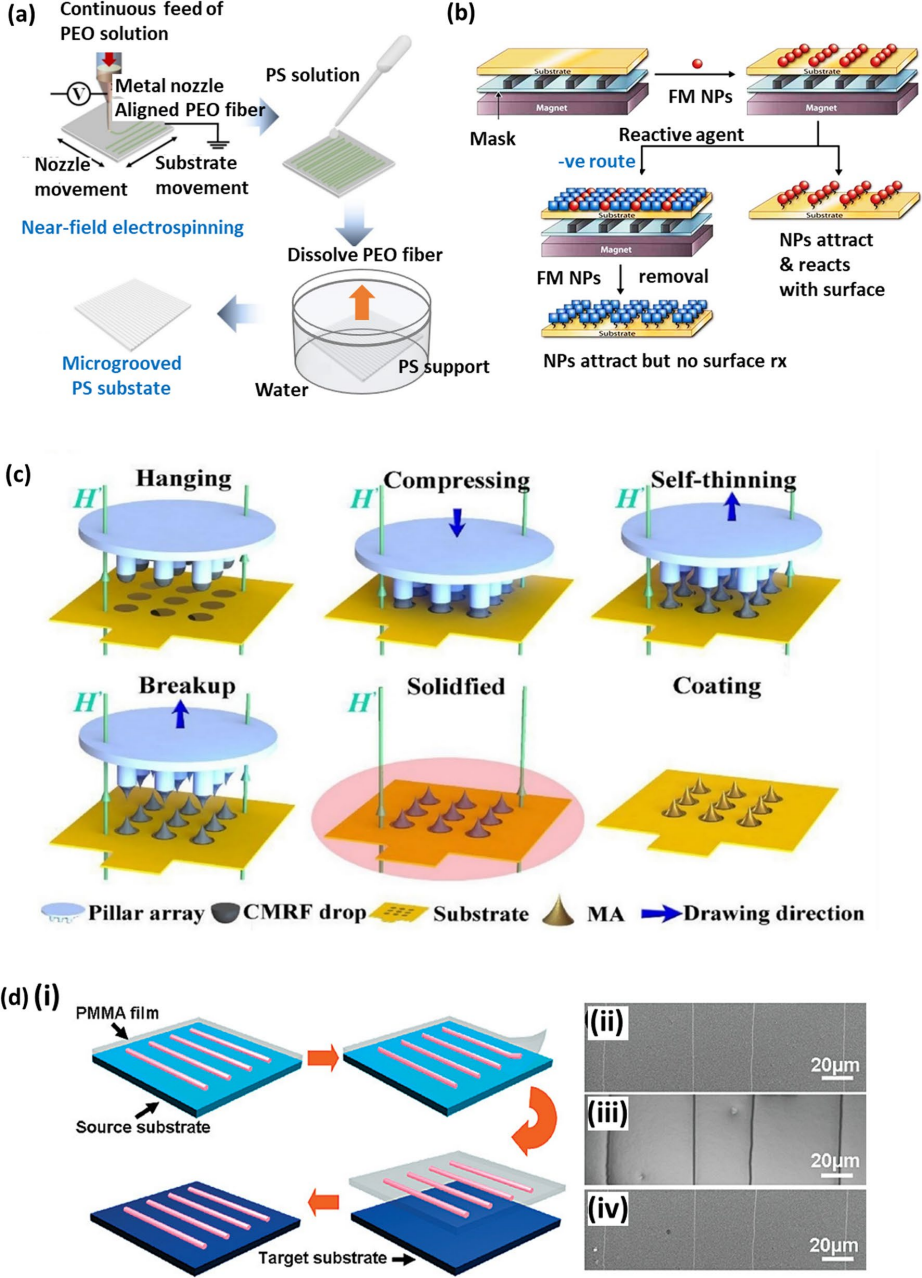
**a** Preparation of micro-grooved polymeric substrate using near-field electrospinning-assisted lithography (NFEAL). Image from [293]. **b** Scheme of two approaches using positive and negative magneto-lithography. The magnetic mask installed at the backside of the substrate and the magnet that is set under the mask induce a magnetic field toward the substrate through the pattern of the masks. In the positive approach, the magnetic nanoparticles (NPs) react chemically with the substrate. Hence, the magnetic NPs are immobilized on selective locations, where the mask induces a magnetic field, resulting in patterned substrates. In the negative approach, the magnetic NPs are inert to the substrate. Hence, once they pattern the substrate, they block their binding site on the substrate from reacting with another reacting agent. After the adsorption of the reacting agent, the NPs are removed, resulting in a negatively patterned substrate. Image from [297]. **c** Fabrication process of FMAE (flexible microneedle array electrode). Image from [307]. **d (i)** Illustration of the procedures of PMMA-mediated nanotransfer printing technique. Spin-coating a PMMA film on source substrate, peeling off the film from substrate, attaching the film to target substrate, and finally removing the mediator. SEM images of SWNT array **(ii)** on SiO2/Si substrate before transfer, **(iii)** embedded in PMMA film, and **(iv)** transferred to another SiO_2_/Si substrate. Image from [346]

Near-field electrospinning-assisted lithography (NFEAL) is a technique employed in the manufacture of microgrooves within polymer scaffolds (Fig. 4a) [293]. Near-Field Electrospinning (NFES) is the first step of NFEAL and involves wetting a tip with a polymer solution. Applying a high voltage between the tip and the substrate produces an electric field, and generates electrostatic forces, creating and elongating a polymer jet to produce fibres which are dropped upon the substrate [294]. The main advantage of NFES is that it reduces the working distance in comparison to conventional techniques, leading to a decrease in the voltage required, thereby enabling a higher single fibre deposition accuracy [295]. Once the polymer fibres have been formed, a scaffold material is deposited onto the substrate. The fibres can then be eradicated via a solvent, leaving microgrooves within the scaffold, the dimensions of which depend on the size of the polymer fibres. By placing the substrate on a stage, different areas of the substrate can be patterned by the movement of the stage.

NFEAL is a simple, efficient and low-cost technique which can fabricate high-resolution microgrooves with widths between 1 and 10 µm and nanometre-scale depths, down to approximately 65 nm. However, difficulties arise when producing large inter-groove distances due to the stage vibration when moving from one target area to another, and so optimising the precision of the stage movement is essential for larger areas to be patterned. Furthermore, although there are standard polymers and solvents used for NFEAL, for example depositing polyethylene oxide fibres which can be removed from a polystyrene scaffold with water as a solvent, challenges may arise from finding a solvent that can remove the fibres produced from different polymers without damaging the scaffold or the substrate.

NFEAL is a relatively new technique and therefore has not been widely used. However, producing micro- and nanogrooves with NFEAL is significantly cheaper, less time-consuming and requires less harmful solvents than conventional techniques such as EBL and PL, and can produce nanometre resolutions, unlike 3D printing. Yang et al. demonstrated that scaffolds produced with NFEAL were able to elongate and align C2C12 cells and could be used in biomedical applications such as tissue engineering and biosensing [293]. Enhancing NFEAL for biomedical applications requires improvements in large-area patterning. Current challenges include difficulty in achieving large-area patterning when reducing the distance between microgrooves, attributed to the vibration of the translation stage during changes in movement direction. Further reduction of this distance is feasible with a higher-precision motion stage system, though this might escalate the initial system cost [293]. Additionally, the patterning of biocompatible polymers, such as polycaprolactone, requires further exploration. Current issues involve low viscosity solutions forming beads and discontinuous lines, hindering the attainment of the desired structure [296].

### Magnetolithography

Magnetolithography (ML) utilises a constant magnetic field, paramagnetic materials, and ferromagnetic nanoparticles to create the desired pattern on a substrate (Fig. 4b) [297]. A mask consisting of a paramagnetic metal is placed on the underside of a substrate whilst in the presence of an external magnetic field, and the ferromagnetic nanoparticles are placed on the topside of the substrate, with their positions determined by the magnetic field gradient generated by the paramagnetic mask [298]. The magnetic field produces a force F which acts on each nanoparticle and is dependent on the susceptibility variation between the nanoparticle and its surroundings Δχ, the volume of the nanoparticle V, the flux density B and the permeability of free space *µ*_0_ [299]:

$$\mathrm{F}=\frac{\mathrm{\Delta \chi V}\left(\nabla \cdot \mathrm{B}\right)\mathrm{B}}{{\upmu }_{0}}$$

In positive ML, patterns are produced by nanoparticles adhering to the surface in areas where the mask-induced magnetic field is present [300]. In negative ML, the nanoparticles are physisorbed upon the surface of the substrate and when a different chemical is applied, adsorption to the substrate is only possible in locations where the nanoparticles are absent, as the nanoparticles block the substrate binding sites. The nanoparticles can then be removed from the substrate to form the final pattern [301].

ML is a simple, inexpensive, high throughput technique which can produce patterns over large areas [301, 302]. Unlike methods such as PL, ML allows curved surfaces to be patterned and does not require a photoresist, reducing the chance of substrate contamination. The entire multistep process can be carried out whilst the substrate remains within a solution, which is especially advantageous in biological applications as the environmental conditions can be monitored and controlled. Furthermore, by utilising low nanoparticle concentrations in a non-equilibrium state for short periods, the fabricated pattern can have dimensions smaller than the pattern defined by the mask, achieving resolutions below 100 nm. However, there are issues with ML which must be considered when fabricating a substrate. Energy is the negative integral of the force and systems tend towards existing in their lowest energy state. Therefore, to reduce the energy of the nanoparticles, the system will try to increase the force acting upon them. Since the force on the nanoparticles is proportional to their volume, large groups of nanoparticles tend to bunch together and act as a single body, limiting the resolution of ML. To combat this, the concentration of nanoparticles in the solution and their adsorption time must be carefully controlled. Furthermore, smaller nanoparticles can generate more uniform structures but require a stronger external magnetic field, therefore there must be a balance between the uniformity of the structures and the cost of running the required apparatus [297].

ML is versatile in its applications in conjunction with other techniques for molecular and biomolecular patterning, as well as physical patterning such as in microelectronic processes like etching, deposition, and ion implantation [297, 298, 300, 302]. For example, ML-patterned surfaces have been applied to differentiate bio-relevant molecules depending on their hydrophobic or hydrophilic properties. Kumar et al. achieved this through negative ML and a non-uniform magnetic field such that the concentration of nanoparticles varies across the substrate. One type of molecule was deposited in between the nanoparticles before the nanoparticles are removed and another molecule was then applied, which binds to the areas now available. The surface could therefore be patterned such that it has a hydrophilic centre and as the distance from the centre increases so does its hydrophobicity [298].

Furthermore, Bardea et al. demonstrated the patterning within tubes using ML for lab-on-a-chip. The nanoparticles were coated and, using positive ML, a magnetic field was applied such that the nanoparticles were immobilised at selected sites within the microchannel. Negative ML can then be used to apply chemicals to the remaining exposed areas. This allowed local and sequential reactions to occur within the microchannels, improving the efficiency and cost of lab-on-a-chip devices [300].

Moreover, the sensitivity of electrochemiluminescence (ECL) immunosensors can be enhanced by utilising magnetic nanoparticles to immobilise primary antibodies. Consequently, ML, with its high throughput and spatial fidelity, provides a straightforward method for depositing magnetic nanoparticles onto a surface and improving immunosensor performance. Studies conducted by Liao et al. and Huang et al. employed ML to create ECL immunosensors for detecting human serum albumin and the epithelial cancer biomarker EpCAM, respectively, showcasing the potential applications of ML in disease monitoring and diagnosis [303, 304].

While magnetic nanoparticles exhibit promising properties for biomedical applications, they face certain limitations, including aggregation, poor storage stability, oxidation and the loss of their magnetic properties. These challenges can be addressed through stabilisation techniques such as, coating with biocompatible inorganic and organic materials [305]. Additionally, magnetic nanoparticles can possess inherent toxicity, but functionalising them with compounds like ligands may enhance their biocompatibility [306]. Despite the potential of magnetic lithography in biomedical applications, the technique is still in its early stages and warrants further exploration.

### Magnetorheological drawing lithography

Magnetorheological drawing lithography (MRDL) is a maskless technique capable of producing microneedle arrays (MAs) on a flexible substrate using a curable magnetorheological fluid (CMRF) and an external magnetic field. The fabrication of flexible microneedle arrays (FMAEs) through MRDL is demonstrated in Fig. 4c [307]. Droplets of the CMRF are suspended from an array of pillars and are subsequently pressed onto the substrate. When the pillar array rises, the CMRF forms liquid bridges which are governed by the forces involved such as the magnetic forces, surface tension, viscous forces, gravity and tensile forces. The liquid bridges thin and grow upwards at their centres, causing instabilities which fracture the liquid bridge, resulting in sharp liquid tips being present on the substrate. The balance of forces and the magnetic field interacting with the iron particles in the CMRF maintains the equilibrium shape of the MAs. The microneedles previously produced were between 600 and 700 µm tall, with a base width of 185–650 µm and a tip width of 10–15 µm [307–309]. MRDL is a cost-effective, simple and rapid technique, making it ideal for mass production [310]. Furthermore, the external magnetic field can be easily changed to direct the growth of the microneedles. However, the microneedles must consist of a magnetic material and, as iron particles are toxic and are present in many CMRFs, the applications of these microneedles are limited [311].

Ren et al. proposed that MRDL could be implemented in the development of certain wearable and flexible sensors, used in measurement and bio-signal monitoring of electrocardiography (ECG), electroencephalography (EEG), and electromyography (EMG) for early detection of conditions such as epilepsy, cardiovascular issues and muscular dystrophy. Unlike other lithographic methods, MAs produced with MRDL did not encounter drawbacks such as rigidity of the substrate preventing full contact with curved human skin, complex and expensive equipment, production of toxic waste and clean room requirements [312]. Furthermore, Gao et al. developed a biosensor system containing a microneedle electrode array with a multi-channel portable electrochemical analyser for the detection of levels of multiple analytes such as glucose, cholesterol and uric acid, which can give indications of the onset of certain conditions such as hypertension, hyperglycaemia, diabetes, and many more, especially in out-of-clinic settings [309]. Chen et al. also demonstrated that MRDL can be used to fabricate microneedles, MAs and dissolvable MA templates for ex vivo transdermal drug delivery for compounds such as insulin, RNA and vaccines [110, 112].

The applications of magnetic resonance drawing lithography are restricted to structures that necessitate microneedles, thereby limiting their versatility. Additionally, thorough investigation into the biocompatibility of the magnetic materials used in microneedles is imperative before their integration into biomedical applications can be realised [313].

### Nanotransfer printing

Nanotransfer printing (NTP) is a simple fabrication technique that explores the controlled arrangement and vertical growth of one-dimensional nanostructures on both soft and hard substrates for various applications (Fig. 4d) [314]. The technique generally involves the transfer of patterns to the target substrate, such as transferring a chosen pattern from a polymeric stamp to another substrate. The structures are first deposited onto a surface known as a donor substrate, a stamp is placed in contact with it and then it is lifted from the donor substrate with the pattern upon it. The stamp is subsequently placed onto a receiving substrate and then slowly removed, finally moving the pattern from the stamp to the receiving substrate [248].

NTP can be used to produce both 2D and 3D nanostructures at a low cost, high throughput and can generate patterns on various substrates [315, 316]. By combining this technique with directed self-assembly or applying heat, resolutions below 10 nm and 20 nm have been attained respectively [317, 318]. However, NTP is a time-consuming process and can only be carried out over small areas [319].

Loo et al. used NTP to create gold contacts on 1,8-octanedithiol/gallium arsenide junctions. Difficulties arise when placing organic materials in electric contact with electrodes due to their lack of robustness. However, NTP demonstrated the ability to produce electric contacts for molecular electronics in a fast and cost-efficient manner in ambient conditions [320]. Furthermore, Hwang et al. used NTP to create large-area transmission-type flexible plasmonic colour filters. Initially, a polyethylene terephthalate (PET) film was attached to a silicon master with pre-patterned nano-sized concavities using EBL. Following a curing procedure and removal of the silicon master, a PET replica made of dome nanostructures was formed. Another PET film was made with the same procedure but without a pattern, and an adhesive layer was spin-coated onto this. Aluminium was then deposited onto the PET replica film and pressed against the surface-treated PET film under heat to adhere the aluminium nanostructures to the flexible PET substrate. NTP provided a fabrication method that was simple and easy to scale up, allowing pattern fabrication over areas of approximately 5 cm^2^ [321]. NTP was also used by Kim et al. to create gold cone-shaped nanostructures for field-emission displays. Initially, the authors patterned a substrate using PL to create negative cone-shaped features and replicated it onto a poly-urethane–acrylate (PUA) mould. Following treatment with a self-assembled monolayer containing HS groups, a layer of gold was deposited onto these structures. Finally, the indium tin oxide-coated glass substrate was cleaned and pressed against the PUA mould. The formation of Au–S bonds chemically binds the gold nano-cone structures to the surface of the coated glass. NTP was advantageous for this work due to its ability to produce metallic nanostructures of any shape in specific locations [12].

Recent advancements in biomedical applications have been made with NTP. Localised plasmon resonance-based biosensors face limitations in widespread adoption, as traditional techniques like EBL and FIBL struggle to fabricate well-defined periodic arrays of metallic nanostructures over large areas at a low cost. Shin et al. and Gao et al. showcased the potential of NTP to overcome these challenges, demonstrating its capability to create gold nanohole arrays, nanodisk arrays, and nanomeshes for plasmonic biosensors [322, 323]. Furthermore, Ko et al. utilised NTP to manufacture smart contact lenses. In contrast to many conventional lithographic methods like EBL and EUVL, NTP is biocompatible. Therefore, it was employed to generate silver nanopatterns on hyaluronic acid (HA) films, producing contact lenses with unique optical characteristics. For example, these lenses could correct dyslexia in patients with Irlen syndrome, caused by hypersensitive reactions to certain colours, and enable the production of augmented reality contact lenses [324].

Similar to other stamp-based techniques *e.g.,* CFL and NIL, the resolution and reproducibility of nano-transfer printing are constrained by the adhesion between the stamp and the substrate, posing limitations on its applicability for the mass production of biomedical devices. While this challenge can be addressed through additional, the potential negative impact on biological components, increased complexity and reduced material compatibility must be considered [315]. However, significant reduction of the adhesion force can have detrimental effects on pattern transfer from the stamp to the substrate, necessitating the development of methods to enhance the fidelity of NTP for its application within the biomedical industry [325].

## Further application areas

**Fig. 5:**
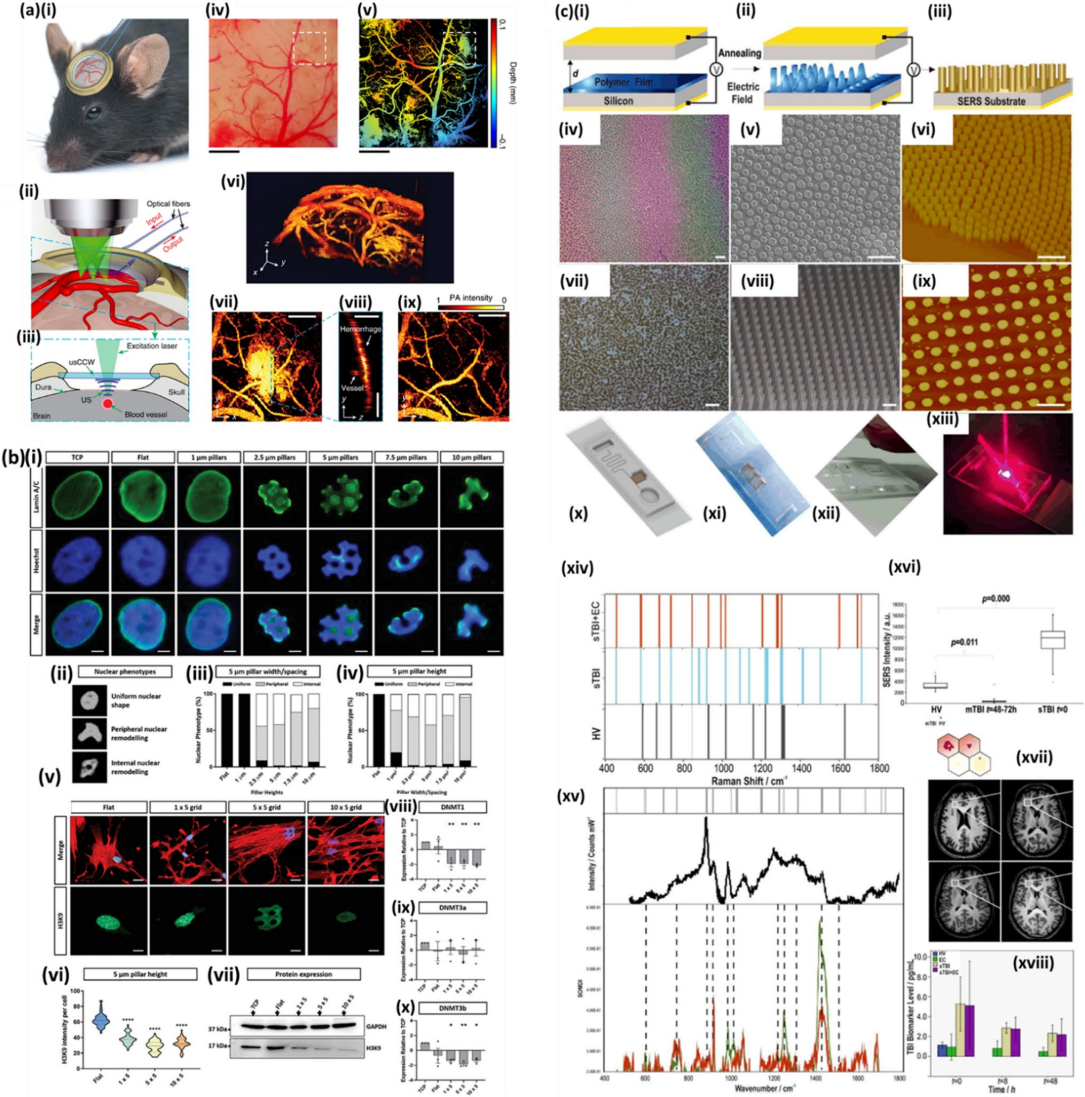
Emerging Lithographies: Recent applications in medicine. **a In-vivo** photoacoustic microscopy (PAM) cortical imaging using an ultrasonic chronic cranial window (usCCW). **(i)** The usCCW is surgically implanted on the mouse skull after craniotomy. The inset shows the physical dimension of the usCCW with micro-ring resonator (MRR), fabricated via nanoimprint lithography, and fibers attached, which is optically transparent, with a total thickness of 250 µm and a total weight of less than 1 g. The MRR ultrasonic detector is attached on an 8-mm diameter circular substrate and the sensing light is coupled through a pair of 30-cm flexible optical fibre. **(ii)** Illustration of optical scanning through the usCCW. To excite the MRR resonance, a narrow-band continuous-wave tuneable laser (New Focus, TLB-6712, wavelength from 765 to 781 nm) is coupled into the bus waveguide after passing through a fibre polarization controller and collected by a multimode fibre on the other end of the bus waveguide. **(iii)** Optical excitation and ultrasonic detection geometry along the cross section highlighted in **(ii)** The space between the MRR and the dura is 1 mm and is filled with 0.5% agarose gel. We seal the usCCW with dental cement to prevent infection and leakage. d Brightfield optical microscopy image of the cortical region through the MRR. **(v)** Depth-encoded maximum-intensity-projection (MIP) PAM image of the same area. The whole image is stitched from 9 acquisitions due to the limited laser-scanning field of view. **(vi)** Three-dimensional visualization of the vessel orientations and cortical curvature. **(vii)** PAM image of the haemorrhage area highlighted by the dashed box in **(iv)** and **(v)**. **(viii)** PAM B-scan image from the position highlight by the green dashed line in g, showing the hidden vessels beneath the haemorrhage area. **(ix)** Visualizing vessels beneath the haemorrhage layer. Scale bars, **(i–ii)** 0.5 mm and **(vii-ix)** 200 µm. Permission from Li [261]. **b.** Micropattern-defined nuclear indentation regulates heterochromatin expression and DNA methyltransferase (DNMT) activity. **(i)** Representative lamin A/C (green) and nuclei (blue) staining demonstrating phenotypes resulting from varied nanoimprinted -patterned spacing and width. Scale bar, 5 µm. **(ii)** Nuclear phenotype categories and associated quantification with changing **(iii)** micropillar height or **(iv)** micropillar width/spacing. **(v)** Fluorescence staining of actin (red), nuclei (blue), and H3K9 (heterochromatin marker (green)). Scale bar, 5 µm. **(vi)** Mean fluorescent intensity quantification of H3K9 expression with changing micropillar width and spacing. **(vii)** Western blotting of H3K9 in MSCs cultured on control substrates and micropatterns with a constant height of 5 µm. **(viii, ix, x)** RT-PCR analysis of DNMT1, DNMT3a, and DNMT3b, respectively for each pattern at a constant 5 µm micropillar height. All graphs show mean ± SD for three independent MSC donors relative to TCP samples. Samples were analysed by one-way ANOVA with Tukey post hoc testing. Statistically different samples are denoted by *p < 0.05, **p < 0.01, and ****p < 0.001. Permission from Carthew [262]. **c** Electrohydrodynamic patterning is based on depositing a thin nanofilm in a capacitor-like device **(i)** and subsequently applying a small voltage, which results in the destabilization of the smooth film and reorganization of the fluid material in the direction of the generated electric filed lines, perpendicular to the substrate, towards the top electrode **(ii)**. Covering with a thin gold layer yields the RED-SERS substrates **(iii)**. Optical microscopy **(iv)** and scanning electron microscopy **(v)** images of electrohydrodynamically patterned pillars under homogeneous electric field. Atomic force microscopy 3D cross sectional image **(vi),** optical microscopy **(vii),** scanning electron microscopy **(viii)** and atomic force microscopy top view **(ix)** images of the uniform EHD-SERS structured substrates. Schematics (vii) of the integrated optofluidic device with the corresponding **(x–xii)** fabricated lab-on-a-chip and the **(xiii)** optofluidic EHD-SERS chip used for the detection during the excitation with the 785 nm laser. Scale bar (i) and (iv): 10 µm, (ii–iii) and (v, vi): 5 µm. **(xiv)** Barcode derived from SERS spectra for (mild/severe) traumatic brain injury (m/sTBI) diagnostics. **(xv)** Top: Representative significant peaks highlighted with vertical grey lines, highlighting the correspondence or the absence of the N-acetylasparate (NAA) peaks with some vibrational frequencies of the bands being unchanged in SERS spectra whereas several are red-shifted or not evident in the healthy control (HV) spectrum. Bottom: Self-organising map (discriminant index) (SOMDI) method applied to the data showing high SOMDI score associated with wavenumbers that strongly influence clustering. **(xvi)** Two-sided statistical analysis of each group and the corresponding SOM (below) demonstrating classification of mTBI versus HV group. Box plots of the plasma biomarker levels in HV, mTBI and sTBI groups, representing the minima, maxima, interquartile ranges, whiskers and the median. NAA levels detected in blood of the two different groups of TBI including, the mTBI at 48–72 h and sTBI at t = 0 and compared with the HV exhibited a different and significant trend. While the NAA was found to be remarkably decreased in mTBI patients compared with HVs, an opposite trend was presented by sTBI patients, showing a significant increase compared to HVs group. **(xvii)** Representative MRI images of concussed athletes (n = 4, out of a total of n = 43). Superior frontal white matter region of interest illustrated. Short echo (35 ms) spectra emphasise improved resolution of lower concentration spectral components. **(xviii)** The optofluidic SERS device data measured for sTBI samples with the TBI indicative markers concentration was significantly higher in sTBI and sTBI + extracranial injury (EC) patients at t = 0 compared with HV. Concentration was also significantly higher in sTBI and sTBI + EC compared with EC at t = 8 h and EC at t = 48 h. Permission from Goldberg Oppenheimer [347]

This review has focused predominantly on biomedical applications of lithographic advancements, where lithography techniques have already found numerous applications (Fig. 5), however, lithography is also impacting other research areas. In closing, we highlight some other emerging application spaces.

### Food safety and security

As food has become an increasingly globalised, industry food supply chains have become complex, in turn leading to more nodes where criminal actors can interfere with foodstuffs for economic gain. This has led to a drive for the development and testing of portable sensing devices that can be taken to processing factories or to the point of harvest for in-the-field analysis. For instance, in a device-comparison study, McVey et al. evaluated the analytical performance of lab-based (benchtop) and portable near-infrared spectrometers in the context of coriander adulteration. The authors note progress in micro(opto-)electro-mechanicals (M(O)Ems), specifically micromirror and linear variable filter (LVF) technologies, as key to miniature spectrometer developments [329]. While UVL is used for LVF production, research has been conducted in nanoimprint-produced LVFs [330].

Elsewhere, the emerging area of drone technology has been identified as useful for remote crop monitoring [331] permitting rapid, high-resolution analysis of large agricultural regions with LiDAR or hyperspectral sensors. Typically, two broad kinds of drone architecture exist, fixed wing and multi-rotor, the latter design most often associated with hobbyists, however, both have found use in agritech, conferring different benefits in terms of cost, flight speed, range and manoeuvrability [332]. Improved availability of replacement parts in commercial drones has been identified as a bottleneck in the adoption of agricultural drone use in many places [333], and could be tackled with high-resolution, cheaper components produced via a suite of emerging lithographies. Drone technology is one of many potential connected sensors for in-the-field monitoring and is related to new digital technologies within food chains, which require improved data storage media fabrication strategies.

### Magnetic storage media

The advent of ‘big data’ and the need for ever-more data storage capacity, has fuelled continued research into high-density storage media. While early magnetic storage focused on multi-platters *i.e.,* more disks, subsequent efforts have been focused on lowering the bit grain size to increase storage capacity. An emerging technology is heat-assisted magnetic recording (HAMR), which employs a laser to heat smaller disk regions over a nanosecond time scale to lower bit domain size [334] and concurrently, alongside increase in areal bit density, multi-actuator systems have also arisen as a way to speed up access times. In both instances, more economical nanofabrication would be beneficial to offset increased costs pertaining to manufacture complexity.

Alternatively, substrate patterning has also been discussed as a viable method to overcome thermal instability of increasingly small bits, for example with contact lithography, which is a form of PL where a mask is in direct contact with the photoresist [335]. Other patterning strategies have also been proposed, specifically with block co-polymers [336, 337], where the chemistry associated with the annealing process can be used to control feature size [338] and the operating vapour pressure, to control the domain length and morphology [337].

### Defence and law enforcement

As technology progresses, so does the ability to generate counterfeit products. It is, therefore, necessary for anti-forgery technologies to evolve to enable the detection of inauthentic duplications. Both TPL and a combination of holographic lithography with direct laser lithography have been employed to create sub-micron structures, the presence of which enables the differentiation between genuine and counterfeit products [339, 340]. Similarly, genuine products can be identified by QR codes concealed within the product to prevent replication. Colniță et al. developed three-dimensional (3D) QR codes via NIL. Pillars with varying pitches enabled areas of different reflectivity to be generated, improving the identification, traceability and security of the product [341].

The advancement of fabrication techniques has also aided the development of technologies for the defence and intelligence industries. Optical camouflage has also been achieved with EBL, enabling objects to be hidden within an image [342]. When single-band camouflage materials are not optimal due to the development of multispectral complementary detection devices, NIL has been applied to fabricate multiscale hierarchical metasurfaces for the camouflage of microwaves, infrared and dual-band lasers [343]. Furthermore, lithographic devices can assist in the detection of hazardous substances. Gao et* al. *[344] fabricated a flexible wrinkled nanocone PET substrate with colloidal lithography and oxygen plasma etching to detect 2,4,6-Trinitrotoluene (TNT) via SERS.

Liu et al. recently developed a wearable SERS sensor. While conventional wearable sensing technology lacks the ability to detect multiple chemical species simultaneously, the device, based on a bottom-up fibrous gold nano-mesh matrix, maintains sensitivity of an electrical sensor instead via plasmonic enhancement, but with the multiplexing ability of Raman spectroscopy. Moreover, the authors demonstrate minimum performance deficit with repeated deformation, as is required for wearables. Various drugs of abuse such as cocaine and synthetic cannabinoid JWH018 have been identified. MDMA has been detected on different surfaces such as a metal can and keyboard. SERS has a well-known sensitivity-reproducibility trade-off and improved uniformity of signal could be achieved with a soft imprinting lithography approach. Although most closely associated with healthcare applications, wearable technologies are potentially broadly applicable. For instance, the authors also use their device to pick up microplastics, 1% polyethylene microbeads, an increasing concern in environmental monitoring [345].

### Environmental monitoring

Jeong et al. developed a nanotransfer-based technology for high-resolution printing on diverse surfaces, including curved faces, with an initial step using polymers in the fabrication of a master template. The research combines two unique features, the use of co-block polymers to fabricate structures on the order of 8 nm and the use of a PDMS gel pad for improved adhesion control, which causes less perturbation to the imprinted nanostructures. Subsequently, the capability for hydrogen sensing was investigated. A response time, *R*t—the time for 90% of the plateau saturation (resistance and current change ratio)-was found to be 8 s with 0.1% H_2_ in a parallel device-sensor configuration [315].

## Conclusions

Lithography is a vital fabrication technique for applications such as electronics, medical devices and sensors. However, current conventional methods such as photolithography and electron beam lithography have limitations such as their resolution, cost and throughput, which has fuelled research into improving existing techniques and developing new fabrication methods to overcome the limitations of conventional techniques.

Biomedical devices in particular have highly specific requirements, which significantly influence the choice of the fabrication strategy. Foremost among these considerations is the biocompatibility of materials, taking into account the interactions between the device material surfaces and biological specimens, ensuring the absence of toxic residues as well as assessing potential adverse interactions, which could compromise measurement integrity. Moreover, surface materials often must allow for further functionalisation such as for instance, the application of antigens to detect specific antibodies in surface plasmon resonance sensors or Raman reporter molecules in SERS devices. In many cases, a deposited metal layer is involved, prompting careful evaluation of how the base material and structure geometries impact the deposited metal and the chemi-adsorptive properties of its surface. Furthermore, sterilisation is a critical factor for many medical devices and any sterilisation process must not adversely alter material properties. The growing interest in portable medical sensors for use outside clinical settings places emphasis on user-friendly platforms, including lab-on-a-chip devices and flexible materials for wearable healthcare devices. Cost-effective, disposable components are essential, necessitating lithographic methods capable of high throughput with economical materials and minimal environmental impact. Given the complexity of biomedical devices, materials must also be compatible with incorporated optics, electronics and microfluidic components, emphasising the integrative nature of any nanofabricated components.

**Fig. 6:**
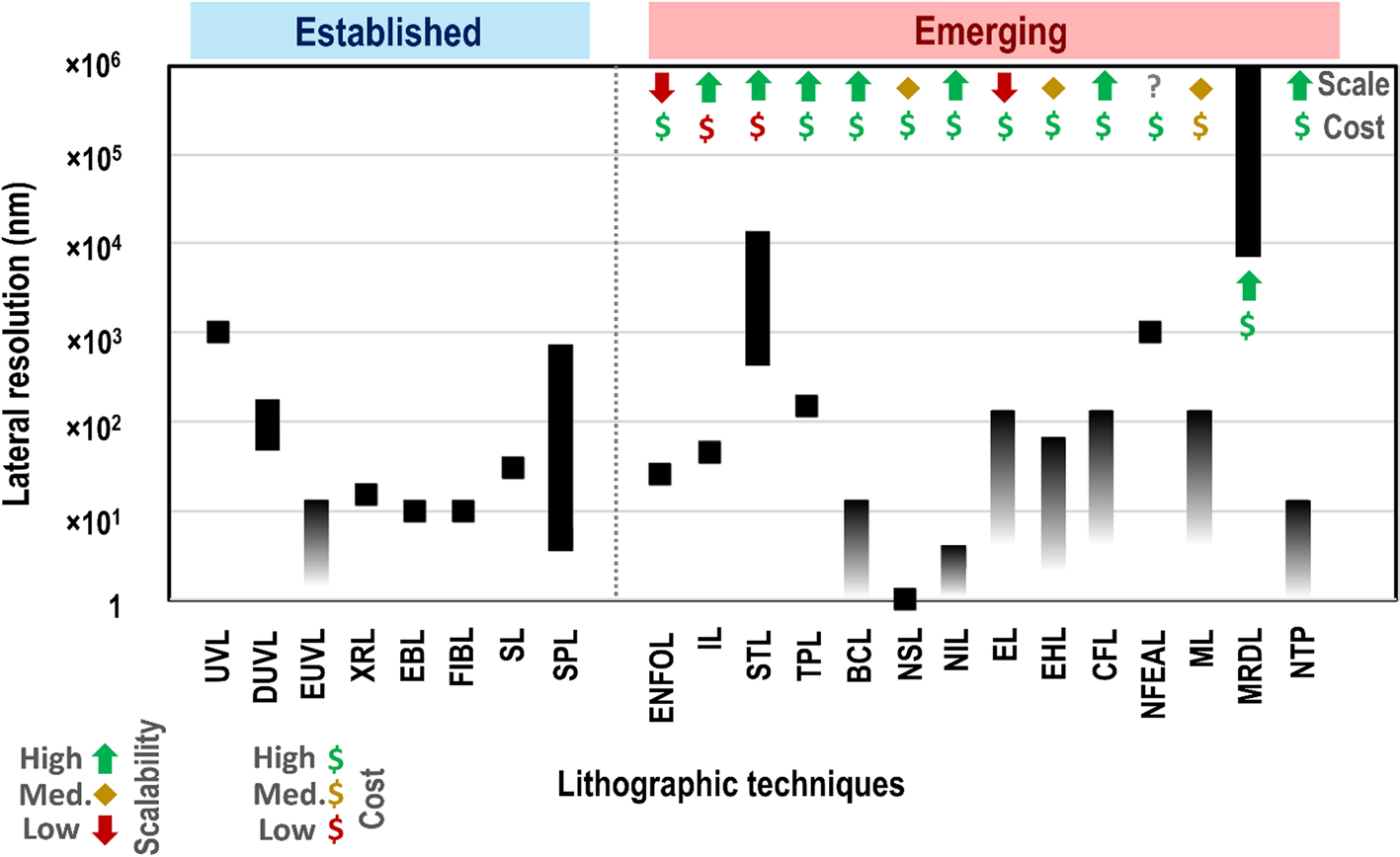
Comparison of established and emerging lithographic techniques with indicators of cost and potential for scalability. UVL = conventional UV lithography, DUVL = deep ultraviolet lithography, EUVL = extreme ultraviolet lithography, XRL = x-ray lithography, EBL = electron beam lithography, FIBL = focused ion beam lithography, SL = soft lithography, SPL = scanning probe lithography, ENFOL = evanescent near-field optical lithography, IL = immersion lithography, STL = stereolithography, TPL = two-photon lithography, BCL = block copolymer lithography, NSL = nanosphere lithography, NIL = nanoimprint lithography, EL = edge lithography, EHL = electrohydrodynamic lithography, CFL = capillary force lithography,, NFEAL = near-field electrospinning-assisted lithography, ML = magnetolithography, MRDL = magnetorheological drawing lithography, NTP = nanotransfer printing

With consideration of the particular requirements for biomedical applications herein, alternative newly emerging lithographic techniques have been discussed including the evanescent near-field optical lithography, immersion lithography, two-photon lithography, stereolithography, electrohydrodynamic lithography, near-field electrospinning-assisted lithography, magneto-lithography, magnetorheological drawing lithography, nanoimprint lithography, capillary force lithography, nanosphere lithography, edge lithography, nanotransfer printing and block copolymer lithography. The strengths and weaknesses of each method have been considered, and several applications in recent literature have been identified. On one hand, some conventional techniques such as extreme UV lithography or X-ray lithography can produce truly nanometric-scale features and are well-understood and scalable but costly, while emerging methods such as, nano-transfer printing or electrohydrodynamic lithography are inexpensive but are yet to become scalable, high-throughput commercialised lithographic techniques. An overall summary of the achievable resolutions, scalability and cost for these conventional and emerging lithographic methods are shown in Fig. 6.

Unsurprisingly, many of the emerging patterning techniques exhibit caveats that may or may not affect the commercial adoption. Nanoimprint lithography for instance, while is low-cost, high-resolution, and potentially scalable, may not always produce high-fidelity patterns, especially at high nanostructure aspect ratios. The suitability therefore will depend on the end-user, whether sensing nanostructures fabricated by NIL, in this example, are intended for qualitative, semi-quantitative, or properly quantitative identification, or whether, for instance, the fine and precise channel formation is essential for a microfluidic device. Moreover, the prevalence of imperfections associated with the various techniques and the steps necessary to avoid defects on a commercial-production scale, and indeed their acceptability and translatability to the end-user, are not yet fully clear. Whilst for instance, NIL could fail to accurately reproduce the sub-master template geometry should the incomplete template depression and air-gaps arise, the emerging technique of nanosphere lithography can often be inhibited due to the dislocations, even on a small scale, whereas the electrohydrodynamic lithography requires precisely spaced capacitor plates over the fabrication region to avoid significant variation in nanostructure dimensions, and co-block polymer lithography requires finely tuned initial conditions to ensure a repeatable outcome. Nevertheless, the advantages of newly developed lithographic techniques have demonstrated considerable potential in the biomedical industry, in applications such as drug delivery, microfluidics, biosensing, cell scaffolds, tissue engineering and biodevices. For instance, the ability of nanotransfer printing to create biocompatible nanostructures over large areas at a low cost has found utility in developing smart contact lenses and localised plasmon resonance-based biosensors and the high reproducibility and fidelity of structures produced under ambient conditions with capillary force lithography has led to its applications in cardiac scaffolds and drug delivery systems. While some of these techniques are still in their early stages, with limited research on their biomedical applications, their inherent advantages hold promise for future development in this industry. Notably, the high resolution and efficiency of immersion lithography make it suitable for manufacturing flat optics for biosensing and biomedical imaging. Similarly, the straightforward and cost-effective production of organic thin-film transistors with electrohydrodynamic lithography could enhance the manufacturing of flexible wearable devices for monitoring, diagnostics and treatment.

Looking forward, beyond specific improvements to individual techniques, several broad research directions are clearly emerging. Many of the lithographic techniques should be applicable to a broad range of materials and research into new materials could improve the commercial amenability. Within nanoimprinting studies for instance, the exploitation of harder PDMS produces more robust templates and higher fidelity structures. Consideration of broadly patternable materials per target application is especially applicable when exploiting the *bottom-up* techniques such as the electrohydrodynamic or block copolymer lithographies, which can integrate technologies across several orders of magnitude using a single process. Alongside this, a continued focus on the accessibility of macroscale 3D structure fabrication, via for instance, stereolithography, is necessary, to accelerate the rapid and affordable device development where there is no longer needed to acquire bespoke components from external vendors. Thus, not only is there plenty more room at the bottom, but maybe a bit more room at the top too. The question whether some of the various lithographic techniques, which are best suited for operation at different length scales, can be combined effectively and subsequently integrated into streamlined workflows, meeting regulations, remains open. Nevertheless, their combination could contribute to appropriate ‘multi-scale’ advanced device developments, where feature sizes spanning from nanoscale up to the macroscale can be simultaneously fabricated.

Overall, scaled-up novel lithographic processes for single-step structuring of large areas will open new avenues for future potential range of applications *e.g.,* intelligent reflective surfaces, microfluidic systems, RF and sonar. Being cost-effective and tuneable, enabling the large-area fabrication of 3D micro and nanostructured platforms, they will combine the capability to directly pattern any material of choice with tuneable dimensions (spanning many length scales) and to tailor-design applied platforms per target application, together with the capability of scaling-up while maintaining cost-effective production. In turn, the newly emerged lithographic techniques for micro and nanostructure fabrication are set to become key enabling processes to support the production of new technologies.

## Data Availability

This is not applicable for this review article
